# Lightweight user authentication scheme for roaming service in GLOMONET with privacy preserving

**DOI:** 10.1371/journal.pone.0247441

**Published:** 2021-02-26

**Authors:** Dongwoo Kang, Hakjun Lee, Youngsook Lee, Dongho Won

**Affiliations:** 1 Department of Electrical and Computer Engineering, Sungkyunkwan University, Suwon, Gyeonggido, Korea; 2 Department of Cyber Security, Howon University, Impi-Myeon, Gunsan-Si, Jeollabuk-Do, Korea; 3 Department of Software, Sungkyunkwan University, Suwon, Gyeonggido, Korea; Wuhan University, CHINA

## Abstract

With the development of information technology and the Internet, users can conveniently use roaming services without time and space restrictions. This roaming service is initiated by establishing a session key between a home node, which exists in a home network, and a mobile node, which exists in a foreign network. However, in the process of verifying a legitimate user and establishing a session key, various security threats and privacy exposure issues can arise. This study demonstrates that the authentication scheme for the roaming service proposed in the existing Global Mobility Network (GLOMONET) environment has several vulnerabilities and, hence, is impractical. In addition, the scheme does not satisfy the privacy of the session key or user’s identity or password. Accordingly, we propose a new lightweight authentication scheme to compensate for these vulnerabilities and secure a high level of privacy, such as non-traceability. In addition, formal and informal analyses are conducted to examine the safety of the proposed scheme. Based on the results of our analyses, we prove that the proposed scheme is highly secure and applicable to the actual GLOMONET environment.

## 1 Introduction

The development of communication technology has provided efficient services based on a sustainable infrastructure, thereby improving the quality of human life. As the scale of the smart device environment increases, we can access various services through our own smart devices and obtain the desired information. In the roaming service provided by the Global Mobile Network (GLOMONET), the mobile node accesses its home node through foreign node roaming not only in the home network, but also in the foreign network [1]. However, because roaming services are provided via public channels, users may be exposed to security threats such as data eavesdropping and location tracking by malicious attackers [2]. Information obtained through eavesdropping is reprocessed by the attacker to guess the user’s identity or password and other sensitive information. Therefore, to guarantee the privacy of users and the secure establishment of a session key, a privacy preserving user authentication scheme is required so that only legitimate mobile nodes can access the home network service through the foreign node. In addition, the same session key must be shared for secure post-communication after the mobile node’s login and follow-up requests have been accepted. Therefore, the session key must not be induced by a malicious attacker. Thus, each participating node must induce the session key through secure information after agreement rather than through direct distribution between nodes.

In this paper, we suggest that the user authentication scheme for GLOMONET, which was recently proposed by Ahmed et al. in 2016, incompletely validates the user’s identity and password and that malicious attackers can guess vulnerabilities. Furthermore, we point out that the existing scheme does not provide traceability of the user. As such, there was a security problem in applying the existing scheme to the actual GLOMONET environment. To compensate for this, we propose a lightweight user authentication scheme for GLOMONET that can completely replace the scheme proposed by Ahmed et al. The proposed scheme follows the security and function requirements of a security and practical user authentication for GLOMONET.

The contributions of this paper are as follows.

1. We analyze the vulnerability of the existing user authentication scheme proposed by Ahmed et al. in the GLOMONET environment, which had not been revealed previously.
2. We propose a lightweight user authentication scheme that uses only a hash function and bit-wise exclusive OR does not use symmetric or asymmetric key encryption, thereby offering both secure roaming services and computation efficiency in the GLOMONET.
3. Our proposed scheme guarantees privacy of the user’s identity and password, anonymity, and even non-traceability, which were not guaranteed by the existing scheme.
4. We demonstrate the safety of our proposed scheme in a formal way using ProVerif and Automated Validation of Internet Security Protocols and Applications (AVISPA), and we used informal analysis to prove that our scheme is successful in preserving privacy and preventing security attacks that can occur in the existing user authentication scheme in GLOMONET.
5. Our proposed scheme can offer expeditious roaming services in the GLOMONET environment with a reasonable computational, communication, smartcard storage overhead.

The remainder of this paper is organized as follows. Section 2 presents related work on user authentication schemes in GLOMONET and the security requirements of user authentication. Section 3 provides preliminary knowledge regarding the authentication process of GLOMONET, the threat model, and the one-way hash function presented in this paper. Then, the authentication scheme presented by Ahmed et al. and its limitations are described in Sections 4 and 5, respectively. Section 6 explains the countermeasures to solve these problems. In Section 7, the secure enhanced GLOMONET user authentication scheme is presented. Sections 8 and 9 prove the safety of the proposed scheme in four ways: formal proof using ProVerif and AVISPA, random oracle model, informal proof, and performance analysis of the proposed scheme. Finally, the conclusions of our study are presented in Section 10.

## 2 Related work

In recent years, there has been considerable research on user authentication schemes for wireless and mobile networks. Inter alia, remote user authentication with a home node via a foreign node on GLOMONET was proposed by Suzuki et al. in 1997 and has since been improved by several researchers [3]. In these networks, privacy preservation is one of the most crucial and assertive tasks. Recently, Yoon et al. developed a new authentication scheme to handle the loopholes of different protocols and argued that this scheme is user-friendly and guarantees the anonymity of the user [4]. However, Niu et al. pointed out that Yoon et al.’s scheme cannot guarantee the anonymity of the user and that the key management system is vulnerable [5]; they then proposed a user authentication scheme based on elliptic curve cryptography (ECC). ECC can employ a relatively short encryption key related to the Rivest-Shamir-Adleman (RSA). ECC uses many wireless devices; therefore, the computing power, memory, and battery life are limited. User authentication scheme–-based ECC has been proposed in the recent year by Li et al. [6] and Chen and Peng [7]. Unlike known encryption methods, Chang et al. and Mun et al. independently proposed a lightweight scheme that used only hash and concatenation functions, which do not use the symmetric key and asymmetric key encryption methods, and improved efficiency by eliminating the need for an “encryption key pre-sharing process” [8, 9]. Subsequently, Gope et al. pointed out that the protocol in Chang et al. is designed on lightweight cryptographic primitives; however, it exchanges eight messages between the participants, which certainly leads to higher communication costs. Nevertheless, the protocols are highly insecure [10]. Likewise, Lee et al. found that the scheme proposed by Mun et al. is not safe from man-in-the-middle and masquerade attacks, and perfect forward secrecy is also not satisfactory [11]. Lee et al. proposed a scheme to solve these security problems, but they highlighted that this scheme is also vulnerable to logical errors and denial-of-service attacks of the registration phase, similar to the scheme proposed by Ahmed et al. Then, Ahmed et al. proposed a new lightweight scheme to address these issues [12]. However, as we mentioned earlier in this subsection, Ahmed et al.’s scheme cannot completely provide user privacy and other security features. As such, according to recent work, a symmetric key encryption method can easily be used to design a user authentication scheme because the security of symmetric key encryption is already guaranteed. However, user authentication schemes using symmetric key encryption methods require pre-key distribution, and the computation cost is significantly slower than the lightweight operation. Conversely, if only the lightweight operation is used, pre-key distribution is not required, and the computation cost is very fast, but the user’s personal information or session key can be easily exposed. Therefore, a secure scheme that can efficiently authenticate users using only a lightweight operation, preserve user privacy, and prevent sensitive information from being leaked would be an ideal for the GLOMONET environment.

## 3 Preliminary knowledge

This section provides preliminary knowledge related to this study. We introduce the overall authentication process in GLOMONET, the applied threat model in the authentication system, the definition and properties of the one-way hash function, and privacy information in user authentication scheme.

### 3.1 Authentication process of GLOMONET

GLOMONET consists of a home node, a foreign node, and a mobile node. The goal of GLOMONET is to provide secure and efficient roaming when the mobile node is out of the network of its home node, sharing the session key and communicating with the home node through the foreign network’s foreign node [13]. This process consists of three major steps.

1. Registration phase: the mobile node registers its identity and password with the home node.
2. Login phase: when the mobile node changes network, the mobile node proves its identity through foreign nodes.
3. Authentication phase: the home node verifies the mobile node and the foreign node, then generates and shares the session key.

**Fig 1:**
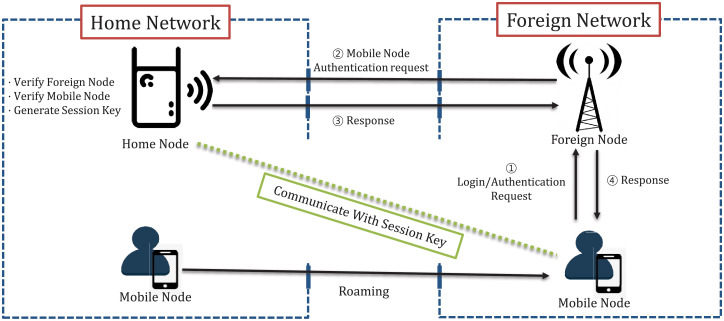
Authentication process of the GLOMONET environment.

The process of a login/authentication phase is illustrated in Fig 1.

### 3.2 Threat model

To analyze the security of the user authentication scheme, we present a threat model based on the Dolev–Yao model [14]. In the threat model, which is applied to both the existing and proposed schemes, the adversary has the following capabilities [15, 16].

1. Any message sent over the public channel can be intercepted by an adversary.
2. An adversary can reprocess, delete, or alter eavesdropping messages.

In addition, in smartcard-based user authentication, we assume that the user’s smartcard is stolen to ensure the safety of the proposed scheme. Thus, the important parameter in it can be acquired via side channel attacks [17]. In addition, an adversary can guess a user’s identity or password offline. According to Wang et al. [18], the dictionary space of a user’s identity or password is 2^20^ ≈ 10^6^, and thus it can be guessed by an adversary within polynomial time.

### 3.3 One-way hash function

A one-way hash function is a function that can be used for data integrity and message authentication. It compresses a string of bits of arbitrary length into a hash code that is a fixed length output. A one-way hash function satisfies the following properties [19, 20].

1. Preimage resistance: if *h*(*x*) is output by the input value *x*, it is computationally impossible to find an input value *x* when only the output value *h*(*x*) is given.
2. Second preimage resistance: given both input *x* and output *h*(*x*), it is computationally impossible to find or produce *x*′ that returns the same *h*(*x*).
3. Collision resistance: it is computationally difficult to find a pair of inputs *x*, *x*′ with the same output *h*(*x*) = *h*(*x*′).

### 3.4 Privacy in the user authentication scheme

In the user authentication scheme, privacy is preserved by satisfying the following four conditions:

1. Parameter privacy: Information related to the user’s identity and password and the session key must not be exposed or derived under any circumstances. In the case of enhanced privacy, the session key is also included in the condition.
2. User anonymity: Even for user verification, the user’s identity should not be exposed as it is in login communication.
3. User non-traceability: Assuming that there are two different sessions, the attacker must not know if they are the same person even though they cannot specify the user of the session.
4. Resistant to user impersonation: Even if the user’s information is not known, an attacker must not be able to trick other parties by pretending to be a legitimate user.

Situational examples where some conditions are not satisfied are described as follows.

**Fig 2:**
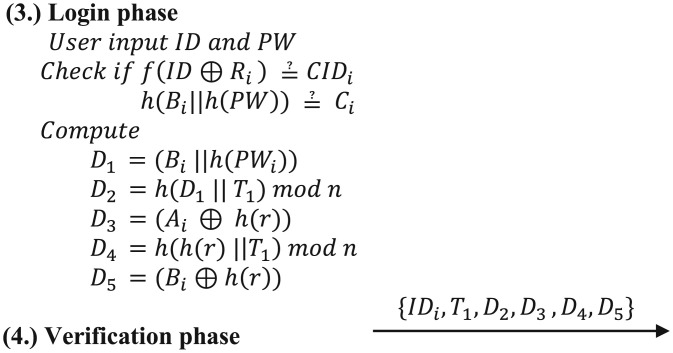
Example of user anonymity not being satisfied.

**Fig 3:**
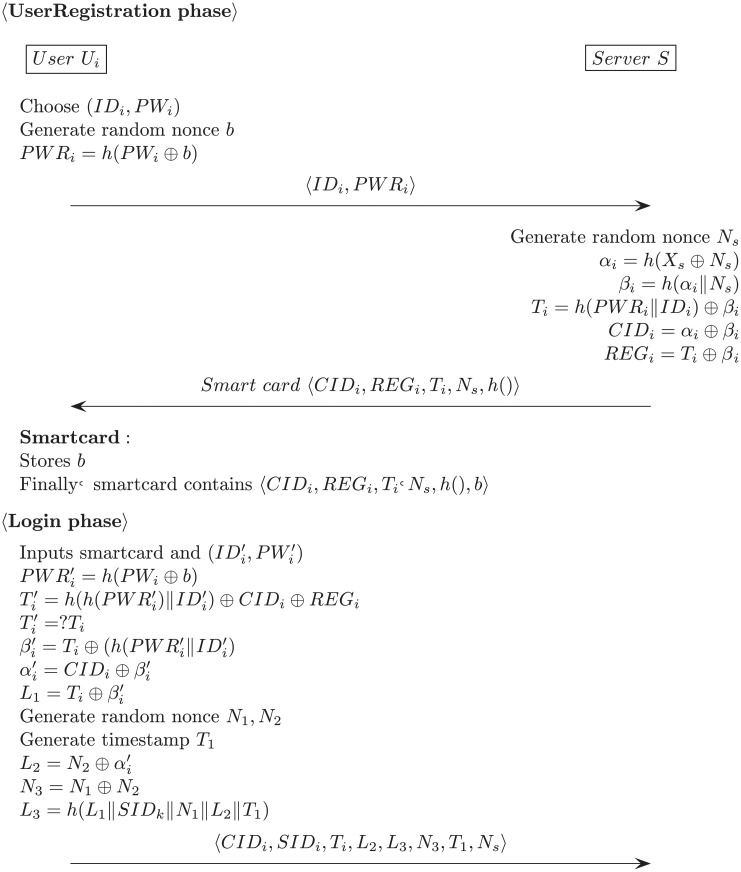
Example of user non-traceability not being satisfied.

Fig 2 presents an example where user anonymity is not satisfied [21]. It can be observed that the user’s identity is transmitted in plain text without any processing during the login phase. Fig 3 presents an example where user non-traceability is not satisfied [22]. The user’s identity and password are hashed with a random number generated by the user during the registration phase in the form of *T*_*i*_ = *h*(*h*(*PW*_*i*_ ⊕ *b*)||*ID*_*i*_) ⊕ *β*_*i*_. In this case, the attacker who steals the login message cannot identify the user. However, the value of *T*_*i*_ is the same for each login attempt; hence, the attacker can determine whether two different login request messages have been generated by the same user through *T*_*i*_.

## 4 Review of Ahmed et al.’s scheme

Ahmed et al.’s scheme for GLOMONET does not use symmetric or asymmetric key encryption, but uses relatively lightweight operations such as bit-wise XOR, hash functions, and concatenation. This section presents the process of Ahmed et al.’s scheme. The scheme consists of four phases: registration, login, authentication, and password change. A guide to the notation is given in Table 1.

**Table 1:** Notation used in Ahmed et al.’s scheme.

Notation	Description
*MN*	Mobile node
*HN*	Home node
*FN*	Foreign node
*h*(.)	One-way hash function
*T*_*x*_	*x*th issued timestamp
⊕	Bit-wise exclusive OR operation
||	Concatenation operation
*R*_*T*_	Registration time
*ID*_*MN*_	Identity of the mobile node
*PW*_*MN*_	Password of the mobile node
*SK*	Session key
*PSK*	Pre-shared key between *FN* and *HN*

### 4.1 Registration phase

(1) The mobile node’s user freely chooses an identity *ID*_*MN*_, password *PW*_*MN*_, and randomly generated nonce *r*.
(2) The mobile node computes *U* = *h*(*PW*_*MN*_||*r*) and sends a registration request message as follows:
  $$\begin{array}{c} \text{Mobile}\;\text{node}\to \text{Home}\;\text{node}:<ID_{MN},U> \end{array}$$
(3) The home node selects a randomly generated random nonce *m*, and computes *B* = *U* ⊕ *h*(*ID*_*MN*_||*m*), *N*_*MN*_ = *h*(*U*||*R*_*T*_) ⊕ *ID*_*MN*_.
(4) The home node issues a smartcard and stores {*B*, *N*_*MN*_, *m*, *U*, *h*(.)}, and then sends it to the mobile node’s user through a secure channel.
(5) The mobile node stores nonce *r* generated in step (1) to the smartcard.

### 4.2 Login phase

(1) The mobile node’s user inputs their identity and password $ID_{MN}^{\prime },PW_{MN}^{\prime }$ into the smartcard.
(2) The node calculates $B^{\prime }=U⊕h(ID_{MN}^{\prime }\left\|m\right)$ then checks $B^{\prime }\overset{?}{=}B$. If not equal, the mobile node terminates the login phase.
(3) The smartcard generates the random nonce *r*_1_ and sends a login request message as follows:
  $$\begin{array}{c} \text{Mobile}\;\text{node}\to \text{Foreign}\;\text{node}:M_{1}=<ID_{HN},K,V,r_{1},T_{1}>\text{where}\\ \; \end{array}$$
  $$\begin{array}{c} K=(N_{MN}⊕U⊕ID_{MN})\\ \; \end{array}$$
  $$\begin{array}{c} SID=h(U\|R_{T})⊕N_{MN}\\ \; \end{array}$$
  $$\begin{array}{c} V=h(K\|SID\|r_{1}\|T_{1})\\ \; \end{array}$$
(4) After receiving the login request message, the foreign node checks the freshness of the timestamp *T*_1_ and *V*, then generates a random nonce *r*_2_ and sends the following message:
  $$\begin{array}{c} \text{Foreign}\;\text{node}\to \text{Home}\;\text{node}:M_{2}=<M_{1},Y,r_{2},T_{2}>\text{where} \end{array}$$
  $$\begin{array}{c} \;\\ Y=h(ID_{FN}\|V\|PSK\|r_{2}\|T_{2})\\ \; \end{array}$$

### 4.3 Authentication phase

(1) After receiving message *M*_2_, the home node checks the freshness of timestamp *T*_2_ and *Y*, *V*, then derives *ID*_*MN*_ = *h*(*U*||*R*_*T*_) ⊕ *N*_*MN*_.
(2) The home node calculates K* = *h*(*ID*_*HN*_||*ID*_*FN*_||*R*_*T*_) and generates session key *SK* = *h*(*ID*_*MN*_||K*||*ID*_*HN*_||*r*_2_).
(3) The home node sends the following message:
  $$\begin{array}{c} \text{Home}\;\text{node}\to \text{Foreign}\;\text{node}:M_{3}=<V_{1},K_{0}.K*,T_{3}>\text{where}\\ \; \end{array}$$
  $$\begin{array}{c} K_{0}=SK⊕h(K*\|V_{1})\\ \; \end{array}$$
  $$\begin{array}{c} V_{1}=h(K*\|ID_{MN}\|K_{0}\|T_{3})\\ \; \end{array}$$
(4) The foreign node checks the freshness of timestamp *T*_3_, derives session key *SK*′ = *K*_0_ ⊕ *h*(K*||*V*_1_), and sends the following message:
  $$\begin{array}{c} \text{Foreign}\;\text{node}\to \text{Mobile}\;\text{node}:M_{4}=<M_{3},r_{2},T_{4}> \end{array}$$
(5) The mobile node checks the freshness of timestamp *T*_4_, calculates $V_{1}^{\prime }=h(K*\|SID\|K_{0}\|T_{3})$, and checks $V_{1}^{\prime }\overset{?}{=}V+1$. If not equal, the mobile node terminates the authentication phase.
(6) The mobile node derives the session key *SK* = *h*(*ID*_*MN*_||K*||*ID*_*HN*_||*r*_2_).

### 4.4 Password change phase

A mobile node user that wants to change their password can request a password change phase.

(1) The user inputs their existing identity $ID_{MN}^{\prime }$ and password $PW_{MN}^{\prime }$, then the smartcard computes $U^{\prime }=h(PW_{MN}^{\prime }\left\|r\right)$.
(2) The smartcard checks $U^{\prime }\overset{?}{=}U$. If not equal, the smartcard rejects the password change phase.
(3) The user inputs their new password *PW*_*new*_, then the smartcard computes *B*′ = (*U*′ ⊕ *h*(*PW*_*new*_||*m*)) and $N_{MN}^{\prime }=h(ID_{MN}\|ID_{HN}\|R_{T}⊕U^{\prime })$.
(4) The smartcard replaces *B* with *B*′, *N*_*MN*_ with $N_{MN}^{\prime }$, and *U* with *U*′ in its contents.

## 5 Security weakness and inefficiency of Ahmed et al.’s scheme

### 5.1 Incomplete verification of identity and password

**Fig 4:**
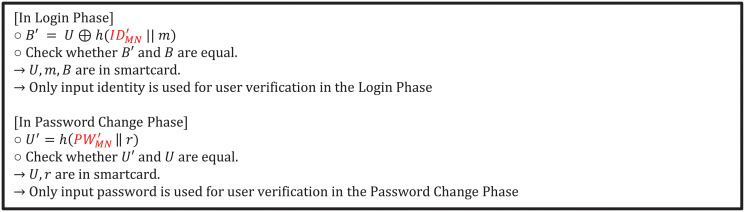
Incomplete verification of identity and password in Ahmed et al.’s scheme.

In a typical authentication scheme, the determination of a user attempting to login begins with verifying that the user has entered the correct identity and password. Also a user wants to change his or her password, the user’s identity and password are first verified and the password change procedure is performed. However, in Ahmed et al.’s scheme, process of verifying the user’s identity and password is incomplete both login and password change phase. In the login phase, user inputs their identity and password $ID_{MN}^{\prime },PW_{MN}^{\prime }$ to login. However, in the login phase, creating *B*′ value with the input identity and password, and if it matches the *B* value stored in the smartcard, it verifies that the user is the legitimate user and proceeds to the next step. However, the only verification that can be done with this comparison is that the stored identity value is the same as the input identity value. Because *B* is composed of *U* ⊕ *h*(*ID*_*MN*_||*m*), and information related to the user’s password(*PW*_*MN*_) is not in *B*. Such that, the verification process succeeds if only the identity is the same even if the user incorrectly enters the password in the login phase. Similarly in the password change phase, user inputs their identity and password $ID_{MN}^{\prime },PW_{MN}^{\prime }$ to password change. Likewise, creating *U*′ value with the input identity and password, and if it matches the *U* value stored in the smartcard, it verifies that the user is the legitimate user and proceeds to the next step. However, the only verification that can be done with this comparison is that the stored password value is the same as the input password value. Because *U* is composed of *h*(*PW*_*MN*_||*r*), and information related to the user’s identity(*ID*_*MN*_) is not in *U*. Therefore, these comparisons cannot properly verify the user. The process of incomplete verification of identity and password in each phase is shown in Fig 4.

### 5.2 Lack of resistance to offline password guessing attack

An offline password guessing attack allows the adversary to guess a user’s password. In Ahmed et al.’s scheme, an adversary that steals a smartcard can use side channel attacks, then simply guess the user’s password using the values in the smartcard. The process of offline password guessing attack is described below.

(1) The adversary extracts *U* and *r* values in smart card through side channel attack (by Section 2.2 Threat model).
(2) The adversary selects the existing password candidate *PW*_1_ through dictionary attack and calculate *U*′ = *h*(*PW*_1_||*r*).
(3) Check whether *U*′ and *U* are equal. If they are different, go back to (2), and pick another password candidate from the dictionary and continue guessing with *PW*_2_, *PW*_3_, …, *PW*_*n*_.
(4) If they are same, the adversary knows that inferred *PW*_*i*_ is the password for the mobile node.

### 5.3 Lack of resistance to offline identity guessing attack

An offline identity guessing attack allows the adversary to guess a user’s identity. In particular, if the identity and password can be guessed independently, the adversary achieves the ideal conditions to impersonate the user. Therefore, an attack that can infer the identity and password should be considered first in a user authentication scheme. In Ahmed et al.’s scheme, *SID* in login phase is same as mobile node’s identity *ID*_*MN*_ for the reason below:

*SID* = *h*(*U*‖*R*_*T*_) ⊕ *N*_*MN*_ [∵ in the Ahmed et al.’s scheme Login phase 4.2 (3)]

⇔ *SID* = *h*(*U*‖*R*_*T*_) ⊕ *h*(*U*‖*R*_*T*_) ⊕ *ID*_*MN*_ [∵ *N*_*MN*_ = *h*(*U*‖*R*_*T*_) ⊕ *ID*_*MN*_ in the Ahmed et al.’s scheme registration phase 4.1 (3)]

⇔ *SID* = *ID*_*MN*_ [∵ *h*(*U*‖*R*_*T*_) ⊕ *h*(*U*‖*R*_*T*_) = 0]

The process of the adversary performing an offline identity guessing attack is described below.

(1) The adversary eavesdropped *V* = *h*(*K*‖*SID*‖*r*_1_‖*T*_1_) in login request message *M*_1_ which communicates via public channel.
(2) According to the above proof, since the *SID* and the identity of the mobile node *ID*_*MN*_ are the same, *V* can be expressed as *V* = *h*(*K*‖*ID*_*MN*_‖*r*_1_‖*T*_1_)
(3) The adversary eavesdropped *K*, *r*_1_, *T*_1_ in login request message.
(4) The adversary selects the existing identity candidate *ID*_1_ through dictionary attack and calculate *V*′ = *h*(*K*‖*ID*_1_‖*r*_1_‖*T*_1_).
(5) Check whether *V*′ and *V* are equal. If they are different, go back to (4), and pick another identity candidate from the dictionary and continue guessing with *ID*_2_, *ID*_3_, …, *ID*_*n*_.
(6) If they are same, the adversary knows that inferred *ID*_*i*_ is the identity for the mobile node.

### 5.4 Absence of mobile node non-traceability

Non-traceability signifies that when the adversary analyzes multiple login request messages, it is impossible to know whether different login request messages are from the same user. Therefore, to satisfy non-traceability, all contents of a login request message should be generated based on a random nonce or the contents of the smartcard should be changed continuously periodically. In Ahmed et al.’s scheme, the login request message container *K* is created using a bit-wise exclusive OR operation of *N*_*MN*_, *U*, and *ID*_*MN*_. However, these three values are never changed after the registration phase. Thus, the value of *K* does not change every time a mobile node user logs in, and non-traceability is not satisfied.

### 5.5 Lack of resistance to mobile node impersonation attack

An impersonation attack implies that an adversary creates a fake login request message for the purpose of passing authentication. Then, the home node receives the message and misinterprets an adversary as a legitimate user through the login phase. The process of impersonating a mobile node and obtaining parameters is described below.

(1) The adversary selects a random nonce $r_{1}^{\prime }$ and current-time-based timestamp $T_{1}^{\prime }$
(2) The adversary calculates *K*′ = *N*_*MN*_ ⊕ *U* ⊕ *ID*_*MN*_ using the smartcard’s container and identity guessing attack (Section 4.3).
(3) The adversary generates $V^{\prime }=h(K^{\prime }\|ID_{MN}\|r_{1}^{\prime }\|T_{1})$.
(4) The adversary sends the fake login request message $M_{1}^{\prime }=<ID_{HN},V^{\prime },K^{\prime },r_{1}^{\prime },T_{1}^{\prime }>$ to the home node via the foreign node.

After the home node receives message $M_{1}^{\prime }$, the home node checks the legitimacy of the mobile node’s message. However, the home node is not able to distinguish between *V*′ and *V* because the adversary has used a legitimate user’s identity *ID*_*MN*_. Thus, the verification phase is terminated normally, and the session key is established with the adversary.

### 5.6 Absence of resistance to session key derived attack

**Fig 5:**
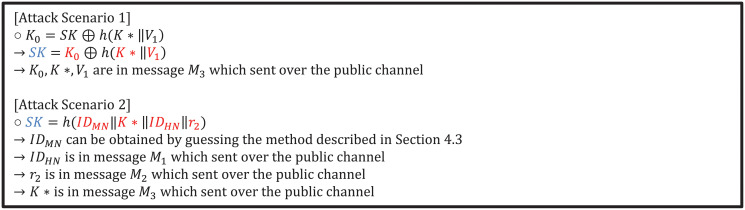
Session key derived attack in Ahmed et al.’s scheme.

The session key is an encryption key used only by one party during a communication session. If there several ciphertexts use one key, the session key may be analyzed to calculate the key. Therefore, the session key should not be derived in any case other than with a legitimate party for communication. However, Ahmed et al.’s scheme allows the adversary to derive a session key and allow the adversary to communicate with the derived session key. In Ahmed et al.’s scheme, the session key can lead to two attack scenarios. Each attack scenario comprises the adversary performing a session key derived attack; these scenarios are shown in Fig 5.

### 5.7 Lack of session key mutual authentication

Mutual authentication means establishing a session key after authenticating a party other than itself to all parties participating in the communication. In addition, the general purpose of mutual authentication is to ensure that the session key created in each party is created correctly. Otherwise, the session key could be tampered with by a malicious adversary, and the session key thus created would be able to communicate with the malicious adversary. However, in Ahmed et al.’s scheme, the session key created in the home node is derived from the mobile node, but the phase ends without any verification of the created session key.

## 6 Countermeasures

In this section, we present a possible mechanism for eliminating the vulnerability in Ahmed et al.’s scheme caused by the lack of user privacy and other security weaknesses. This vulnerability is due to the following reasons.

1. As all information, except the password, is stored on a smartcard, offline password-guessing attacks are possible.
2. All information, except the identity, is also transmitted to the login request message; as a result, offline identity-guessing attacks are possible by eavesdropping the login request message.
3. After the authentication phase is over, information related to the user’s privacy is not updated, resulting in mobile node traceability and impersonation through the previous login request message.
4. The session key should consist only of information shared reliably and safely between parties.
5. There must be a means to verify that the session key generated by each different party is correctly generated.

### 6.1 Secure protection of identity and password

To address the aforementioned weaknesses, first, secure protection of the identity and password is required. Because privacy information, such as a user’s identity or password, can be guessed by an adversary, it is important to make it impossible to infer it solely by combining it with other random nonces. To this end, we protect the password with the random nonce generated in the registration phase; further, the system is designed to require the correct identity to induce a random nonce. In other words, to verify whether the user has entered the correct identity and password, a mutual derivation structure was created that derives a random nonce through the entered identity and verifies the password through the derived random nonce.

#### Example of countermeasure

Create pseudo-identity *CID* using random nonce *n* such that *CID* = *ID* ⊕ *n*. In addition, make pseudo-password *CPW* using the same nonce *n* as *CPW* = *PW* ⊕ *n*. When the user inputs identity *ID*′, *n*′ is derived as *n*′ = *CID* ⊕ *ID*′ through the entered identity, and *CPW* is verified through the derived *n*′ and input password *PW*′. When this method is used, the adversary cannot induce the user’s identity and password unless they first guess the random nonce.

### 6.2 Satisfy user non-traceability

To satisfy the user’s non-traceability, the home node needs to update the user’s information in the authentication phase. The home node generates a random nonce and updates the user’s information with the generated random nonce, and the values in the user’s smartcard must be updated accordingly. In addition, the generated random nonce must be safely transmitted in the public channel, which has the risk of eavesdropping. To this end, the scheme must protect the random nonce with the value that only the user can derive so that the user can safely derive the random nonce created by the home node.

#### Example of countermeasure

Generate random nonce *n*′ on the server. Let us say that *CID* is the user information shared by the existing user and the server. If the user makes the existing *CID* based on random nonce *n*, it updates the CID information, such as *CID*′ = *h*(*CID*||*n*′), and securely sends random nonce *n*′ to the user. The user updates *CID* to *CID*′ using the received random number *n*′. In future login requests, messages are now generated based on *CID*′ instead of *CID*, and thus, the user’s login request message is completely changed, and non-traceability is achieved.

### 6.3 Secure session key and mutual authentication

The session key is also created based on the random nonce created by each mobile node and foreign node, and the scheme is designed to securely protect the generated random nonce. The scheme must also employ various credentials in the session key generation because these parameters can be kept unavailable to attackers, who, as a result, cannot compute a session key within a reasonable time. In addition, it creates an additional variable that can verify that the session key is correct, and this enables mutual authentication for the session key.

#### Example of countermeasure

If the random number created by the user and the server are *n*_1_ *andn*_2_, respectively, it is safe to include *h*(*n*_1_) and *h*(*n*_2_) as the session keys, respectively. If the secure information shared by only the user and the server is *X*, the session key generated by the server is *SK*. Then, calculate the new variable *V* = *h*(*SK*||*X*) and send variable *V* to the user. If the user also creates a session key *SK*′ with the information received from the server, calculate *V*′ = *h*(*SK*′||*X*) using the created *SK*′ and compare the received *V* with the value. If the compared values are the same, it can be verified that the session key has been derived correctly.

All the modifications suggested in Section 6 are combined to propose an improved authentication scheme, which is described in Section 7.

## 7 Proposed scheme

In this section, we propose a user authentication scheme for GLOMONETs that overcomes the limitations of the authentication scheme proposed by Ahmed et al. We propose a lightweight authentication scheme using only bit-wise XOR and hash functions without using encryption algorithms to objectively compare its performance with Ahmed et al.’s scheme. In addition, our proposed scheme consists of four phases: registration, login, authentication, and password change.

### 7.1 Registration phase

**Fig 6:**
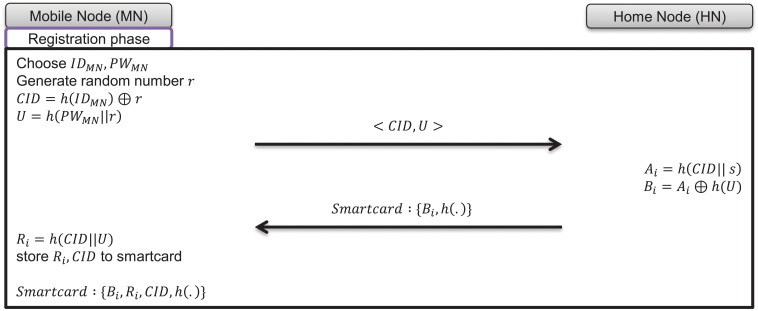
Registration phase of proposed scheme.

In the registration phase, the user of the mobile node selects their identity and password, registers with the home node in its home network, and receives a smartcard from the home node. The progress of the registration phase is as follows and is depicted in Fig 6.

(1) The mobile node’s user freely chooses identity *ID*_*MN*_, password *PW*_*MN*_, and randomly generated nonce *r*.
(2) The mobile node computes *U* = *h*(*PW*_*MN*_||*r*), *CID* = *h*(*ID*_*MN*_||*r*), and sends a registration request message as follows:
  $$\begin{array}{c} \text{Mobile}\;\text{node}\to \text{Home}\;\text{node}:<CID,U> \end{array}$$
(3) The home node computes *A*_*i*_ = *h*(*CID*||*s*), *B*_*i*_ = *A*_*i*_ ⊕ *h*(*U*).
(4) The home node issues a smartcard and stores {*B*_*i*_, *h*(.)}, then sends it to the mobile node’s user through the secure channel.
(5) The mobile node computes *R*_*i*_ = *h*(*CID*||*U*) and stores *R*_*i*_ generated in step (1) to the smartcard.

### 7.2 Login phase

**Fig 7:**
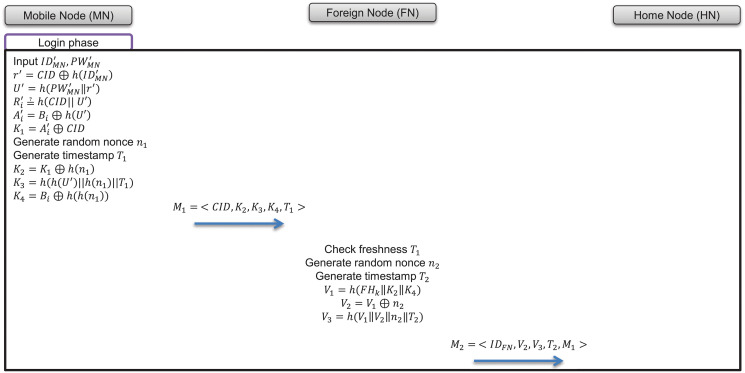
Login phase of proposed scheme.

In the login phase, users who have moved to a foreign network enter their identity and password through a smartcard to use their home network service. Then, the smartcard verifies whether the value is correct by comparing it with the information in the smartcard. Next, the foreign node transmits the user’s information and the foreign node’s information to the home node in the user’s home network. The process of the login phase is as follows and is depicted in Fig 7.

(1) The mobile node’s user inputs identity and password $ID_{MN}^{\prime }$ and $PW_{MN}^{\prime }$, respectively into the smartcard.
(2) The mobile node calculates $r^{\prime }=CID⊕h\left(ID_{MN}^{\prime }\right)$, $U^{\prime }=h(PW_{MN}^{\prime }\|r^{\prime })$, then checks $R_{i}\overset{?}{=}h(CID\|U^{\prime })$. If not equal, the mobile node terminates the login phase.
(3) The smartcard generates a random nonce *n*_1_ and computes $K_{1}=A_{i}^{\prime }⊕CID$. Then, it sends a login request message as follows:
  $$\begin{array}{c} \text{Mobile}\;\text{node}\to \text{Foreign}\;\text{node}:M_{1}=<CID,K_{2},K_{3},K_{4},T_{1}>\text{where}\\ \; \end{array}$$
  $$\begin{array}{c} K_{2}=K_{1}⊕h\left(n_{1}\right)\\ \; \end{array}$$
  $$\begin{array}{c} K_{3}=h(h\left(U^{\prime }\right)\|h\left(n_{1}\right)\|T_{1})\\ \; \end{array}$$
  $$\begin{array}{c} K_{4}=B_{i}⊕h\left(h\left(n_{1}\right)\right)\\ \; \end{array}$$
(4) After receiving a login request message, the foreign node checks the freshness of timestamp *T*_1_, generates a random nonce *n*_2_, and sends the following message:
  $$\begin{array}{c} \text{Foreign}\;\text{node}\to \text{Home}\;\text{node}:M_{2}=<ID_{FN},V_{2},V_{3},T_{2},M_{1}>\text{where} \end{array}$$
  $$\begin{array}{c} \;\\ V_{1}=h(FH_{k}\|K_{2}\|K_{4})\\ \; \end{array}$$
  $$\begin{array}{c} V_{2}=V_{1}⊕n_{2}\\ \; \end{array}$$
  $$\begin{array}{c} V_{3}=h(V_{1}\|V_{2}\|n_{2}\|T_{2}) \end{array}$$

### 7.3 Authentication phase

**Fig 8:**
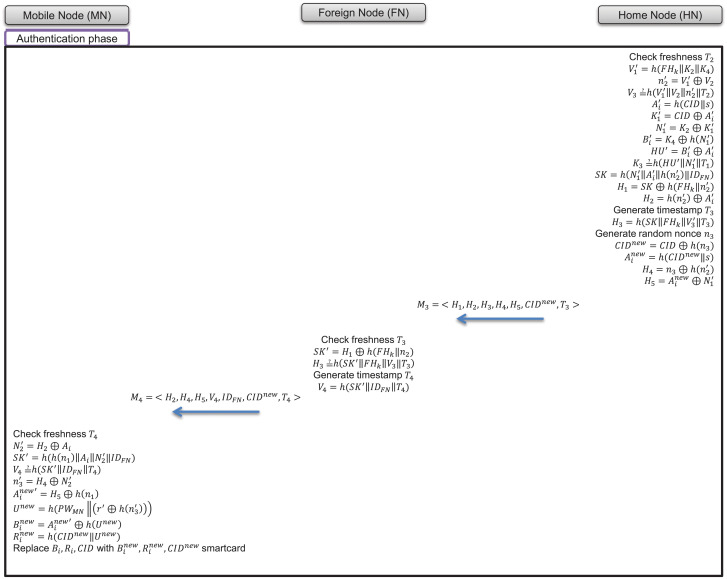
Authentication phase of proposed scheme.

In the authentication phase, the home node uses the message received from the foreign node to verify the validity of the foreign node and the mobile node. Next, a session key is created to start the service with the mobile node in the foreign network, then the session key is sent to the home node via the foreign node. The progress of the login phase is as follows and is depicted in Fig 8.

(1) After receiving message *M*_2_, the home node checks the freshness of timestamp *T*_2_, derives $V_{1}^{\prime }=h(FH_{k}\|K_{2}\|K_{4})$ and $n_{2}^{\prime }=V_{1}^{\prime }⊕V_{2}$, and then checks $V_{3}\overset{?}{=}h(V_{1}^{\prime }\|V_{2}\|n_{2}^{\prime }\|T_{2})$. If not equal, the home node determines that the validity of the foreign node has not been confirmed and terminates the authentication phase.
(2) Then, the home node calculates $A_{i}^{\prime }=h\left(CID\|s\right)$, $K_{1}^{\prime }=CID⊕A_{i}^{\prime }$, $N_{1}^{\prime }=K_{2}⊕K_{1}^{\prime }$, $B_{i}^{\prime }=K_{4}⊕h\left(N_{1}^{\prime }\right)$, and $HU^{\prime }=B_{i}^{\prime }⊕A_{i}^{\prime }$.
(3) The home node checks $K_{3}\overset{?}{=}h(HU^{\prime }\|N_{1}^{\prime }\|T_{1})$; if not equal, the home node determines that the validity of the mobile node has not been confirmed and terminates the authentication phase.
(4) The home node generates a session key as $SK=h(N_{1}^{\prime }\|A_{i}^{\prime }\|h\left(n_{2}^{\prime }\right)\|ID_{FN})$.
(5) The home node generates a random nonce *n*_3_ and sends the following message:
  $$\begin{array}{c} \text{Home}\;\text{node}\to \text{Foreign}\;\text{node}:M_{3}=<H_{1},H_{2},H_{3},H_{4},H_{5},CID^{new},T_{3}>\text{where}\\ \; \end{array}$$
  $$\begin{array}{c} H_{1}=SK⊕h(FH_{k}\|n_{2}^{\prime })\\ \; \end{array}$$
  $$\begin{array}{c} H_{2}=h\left(n_{2}\right)⊕A_{i}^{\prime }\\ \; \end{array}$$
  $$\begin{array}{c} H_{3}=h(SK\|FH_{k}\|V_{3}^{\prime }\|T_{3})\\ \; \end{array}$$
  $$\begin{array}{c} H_{4}=n_{3}⊕h\left(n_{2}^{\prime }\right)\\ \; \end{array}$$
  $$\begin{array}{c} CID^{new}=CID⊕h\left(n_{3}\right)\\ \; \end{array}$$
  $$\begin{array}{c} A_{i}^{new}=h(CID^{new}\left\|s\right)\\ \; \end{array}$$
  $$\begin{array}{c} H_{5}=A_{i}^{new}⊕N_{1}^{\prime }\\ \; \end{array}$$
(6) The foreign node checks the freshness of timestamp *T*_3_, derives a session key *SK*′ = *H*_1_ ⊕ *h*(*FH*_*k*_||*n*_2_), and checks the session key as $H_{3}\overset{?}{=}h(SK^{\prime }\|FH_{k}\|V_{3}\|T_{3})$.
(7) The foreign node sends the following message:
  $$\begin{array}{c} \text{Foreign}\;\text{node}\to \text{Mobile}\;\text{node}:M_{4}=<H_{2},H_{4},H_{5},V_{4},ID_{FN},CID^{new},T_{4}>\text{where}\\ \; \end{array}$$
  $$\begin{array}{c} V_{4}=h(SK^{\prime }\|ID_{FN}\|T_{4})\\ \; \end{array}$$
(8) The mobile node checks the freshness of timestamp *T*_4_ and calculates $N_{2}^{\prime }=H_{2}⊕A_{i}$.
(9) The mobile node derives a session key $SK^{\prime }=h(h\left(n_{1}\right)\|A_{i}\|N_{2}^{\prime }\|ID_{FN})$, then checks $V_{4}\overset{?}{=}h(SK^{\prime }\|ID_{FN}\|T_{4})$. If not equal, the mobile node terminates the authentication phase.
(10) To remove the traceability of the mobile node, the contents of the smartcard are changed as follows:
  $$\begin{array}{c} A_{i}^{\prime new}=H_{5}⊕h\left(n_{1}\right)\\ \; \end{array}$$
  $$\begin{array}{c} n_{3}^{\prime }=H_{4}⊕N_{2}^{\prime }\\ \; \end{array}$$
  $$\begin{array}{c} U^{new}=h(PW_{MN}\|\left(r^{\prime }⊕h\left(n_{3}^{\prime }\right)\right))\\ \; \end{array}$$
  $$\begin{array}{c} B_{i}^{new}=A_{i}^{\prime new}⊕h\left(U^{new}\right)\\ \; \end{array}$$
  $$\begin{array}{c} R_{i}^{new}=h(CID^{new}\|U^{new})\\ \; \end{array}$$
  $$\begin{array}{c} \text{smartcard}\;\text{replaces}\;B_{i},R_{i},CID\;\text{with}\;B_{i}^{new},R_{i}^{new},CID^{new}\\ \; \end{array}$$

### 7.4 Password change phase

**Fig 9:**
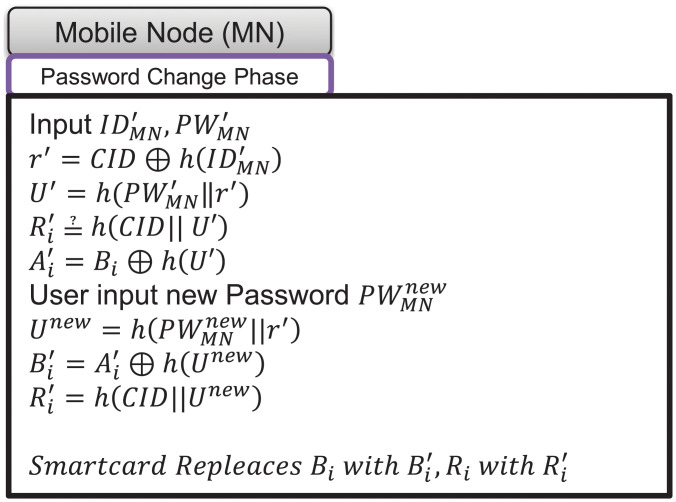
Password change phase of proposed scheme.

If a mobile node’s user wants to change their password, they can request a password change phase. The proposed scheme’s password change phase proceeds as follows. The process of the password change phase is as follows and is depicted in Fig 9.

(1) A user inputs their existing identity $ID_{MN}^{\prime }$ and password $PW_{MN}^{\prime }$, then the smartcard computes $r^{\prime }=CID⊕h\left(ID_{MN}^{\prime }\right)$ and $U^{\prime }=h(PW_{MN}^{\prime }\|r^{\prime })$.
(2) The smartcard checks $R_{i}\overset{?}{=}h(CID\|U^{\prime }))$. If not equal, the smartcard rejects the password change phase. If equal, the smartcard derives $A_{i}^{\prime }=B_{i}⊕h\left(U^{\prime }\right)$.
(3) The user inputs their new password $PW_{MN}^{new}$, then the smartcard computes $U^{new}=h(PW_{MN}^{new}\|r^{\prime })$, $B_{i}^{\prime }=A_{i}^{\prime }⊕h\left(U^{new}\right)$, and $R_{i}^{\prime }=h(CID\|U^{new})$.
(4) The smartcard replaces *B*_*i*_ with $B_{i}^{\prime }$ and *R*_*i*_ with $R_{i}^{\prime }$ in its contents.

## 8 Security analysis

In this section, we use formal and informal methods to demonstrate the safety of our proposed scheme. Formal analysis using ProVerif and AVISPA proves that our proposed scheme performs mutual authentication and securely protects the session key and secret information. In addition, informal analysis proves that our proposed scheme can resist attacks from existing authentication schemes.

### 8.1 Formal security analysis: ProVerif

#### Overview of ProVerif

ProVerif is an automatic cryptographic protocol verifier in the formal model. It is based on a representation of the protocol using Horn clauses and can handle many difference cryptographic primitives, including public-key cryptography, hash functions, and Diffie–Hellman key agreements [23, 24]. In addition, ProVerif can handle an unbounded number of sessions of the protocol and message space. We can prove the following using ProVerif.

1. Secrecy: the adversary cannot compute the considered piece of data.
2. Strong secrecy: the adversary has no information regarding the value of the secret.
3. Authentication: each participant of protocol runs the protocol apparently with another participant.
4. Equivalences: the adversary cannot distinguish between two protocols.

#### Simulation code materialization

Simulation code for verification of our proposed scheme was implemented with eight components.

1. Channels: defines channels used for communication between each party. In total, there are three channels, and *‘cha’* is set as private because it is a secure channel used for communication between mobile node and home node in the registration phase. The channel *‘chb’* is the public channel used for communication between the mobile node and foreign node. The channel *‘chc’* is the public channel used for communication between the foreign node and home node.
2. Constants: defines constants used in the protocol. The identity and password of the mobile node are defined as *‘IDmn’* and *‘PWmn’*. These two constants are set to private because they are confidential information that should not be disclosed, but the attacker was able to guess *‘IDmn’* and *‘PWmn’*, so they were set to weaksecret. The identity of the home node and foreign node are not set to private because they are public information.
3. Secret key: defines a long-term secret key used in the protocol. In our proposed scheme, there are *‘s’*, the secret key of the server, and *‘FHk’*, the pre-shared key between the foreign node and the home node.
4. Shared key: defines the session key derived from the protocol.
5. Functions: defines the function used in the protocol. In our proposed scheme, concatenation, bit-wise exclusive OR, and hash function are defined and each function’s expressions are written.
6. Events: defines the communication start and end events of each node.
7. Process: defines the communication process for each node. Channels and parameters transmitted through channels can be defined through the *‘out’* and *‘in’* functions during the communication process.
8. Queries: defines the query on which the attacker’s capabilities will be modeled and verified. We verify that the session key and the identity and password of the user cannot be derived by an attacker and the internodal relationships are used to determine the process of the proposed scheme in the proper order.

**Fig 10:**
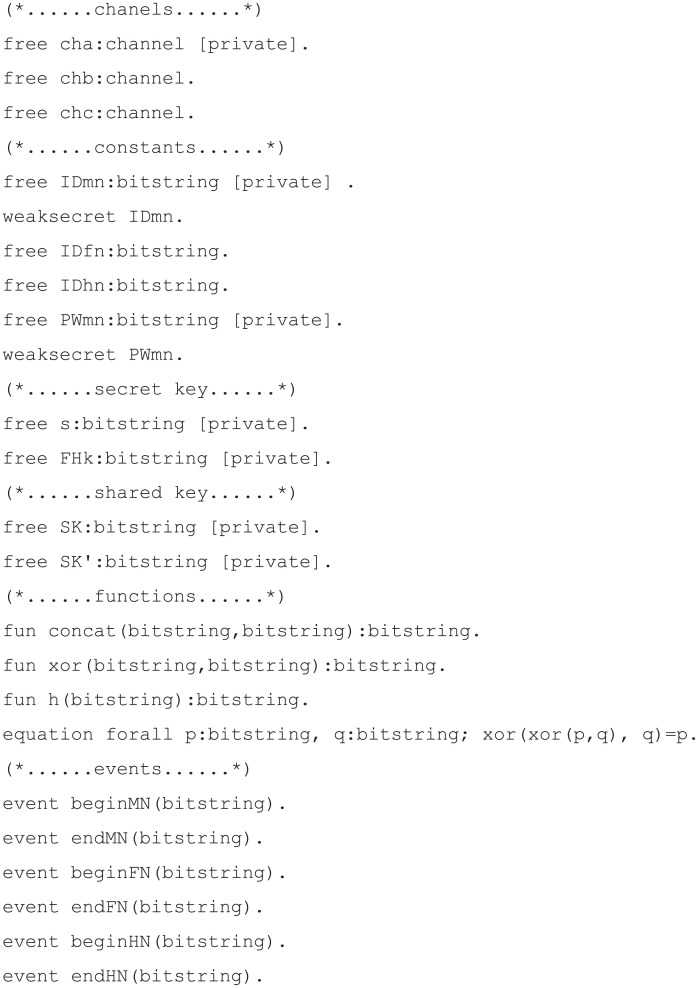
ProVerif simulation code for channels, constants, secret key, shared key, and functions.

**Fig 11:**
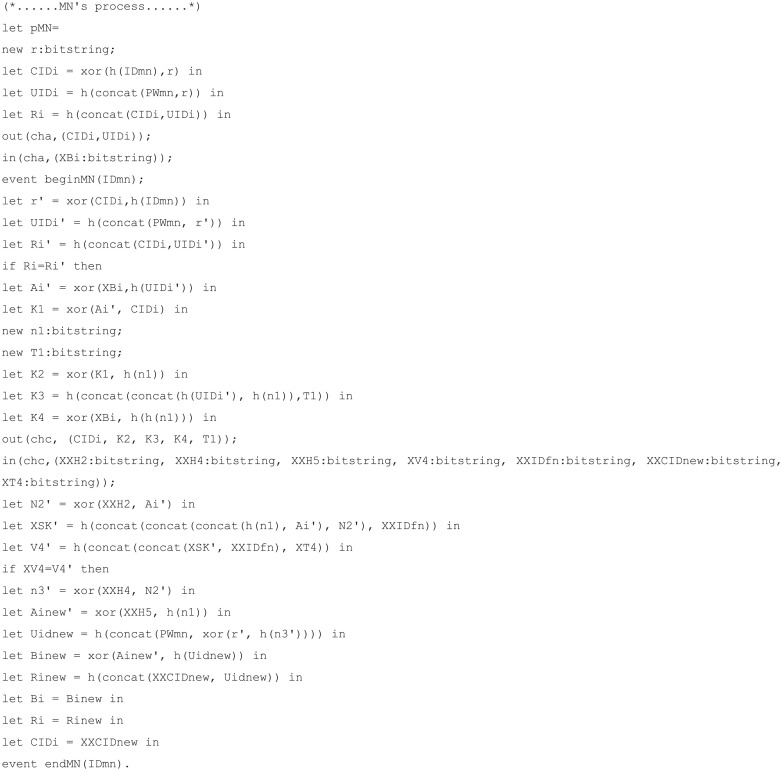
ProVerif simulation code for mobile node process.

**Fig 12:**
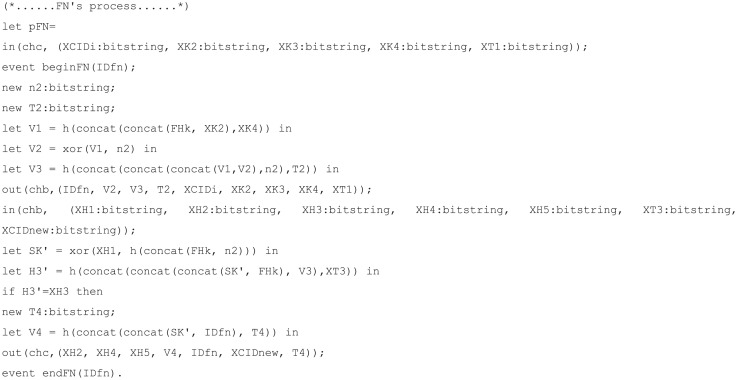
ProVerif simulation code for foreign node process.

**Fig 13:**
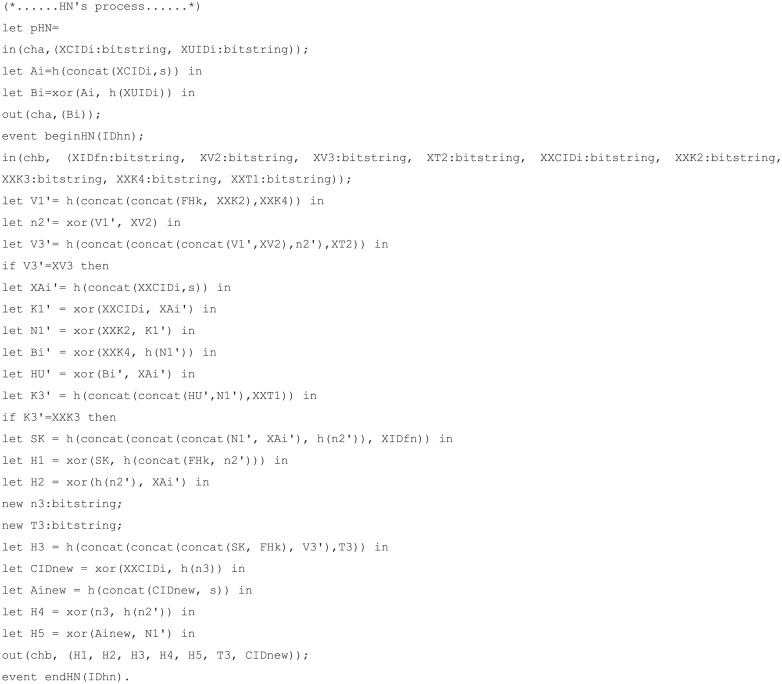
ProVerif simulation code for the home node process.

**Fig 14:**
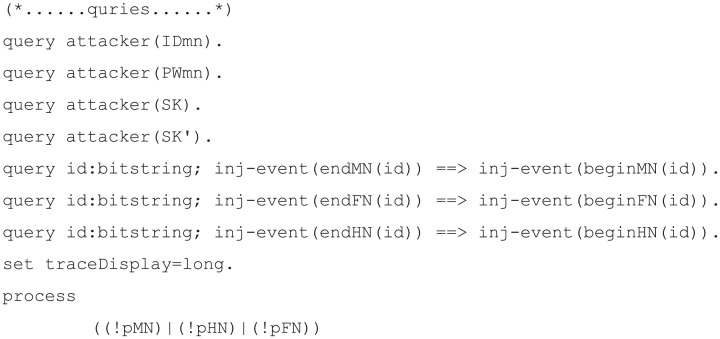
ProVerif simulation code for queries.

The code for channels, constants, secret key, shared key, and functions is displayed in Fig 10; the mobile node, foreign node, and home node process codes for each are displayed in Figs 11, 12, and 13, respectively. The code for query is displayed in Fig 14.

#### Simulation result

ProVerif outputs the results of query as the following five results [25].

- RESULT [Query] is true: the query is proved, there is no attack. In this case, ProVerif displays no attack derivation and no attack trace.
- RESULT [Query] is false: the query is false, ProVerif has discovered an attack against the desired security property.
- RESULT [Query] cannot be proved: ProVerif could not prove that the query is true and also could not find an attack that proves that the query is false.
- RESULT inj-event[Event] ⇒ inj-event[Event] is true: the latter event is proved; that is, the authentication of the former to latter holds.
- RESULT inj-event[Event] ⇒ inj-event[Event] is false: the latter event is not proved; that is, the authentication of the former to latter does not hold.

**Fig 15:**
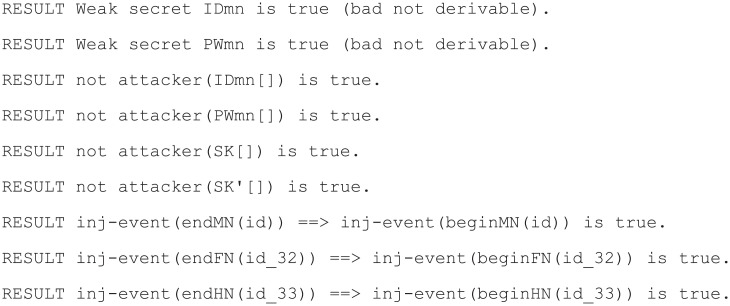
ProVerif simulation result for proposed scheme.

The simulation results of ProVerif for our proposed scheme are displayed in Fig 15.

Thus, our proposed scheme can safely protect the session key and mobile node’s secret information from the attacker. Furthermore, it can be seen that internodal mutual authentication has been achieved.

### 8.2 Formal security analysis: AVISPA

#### Overview of AVISPA

**Fig 16:**
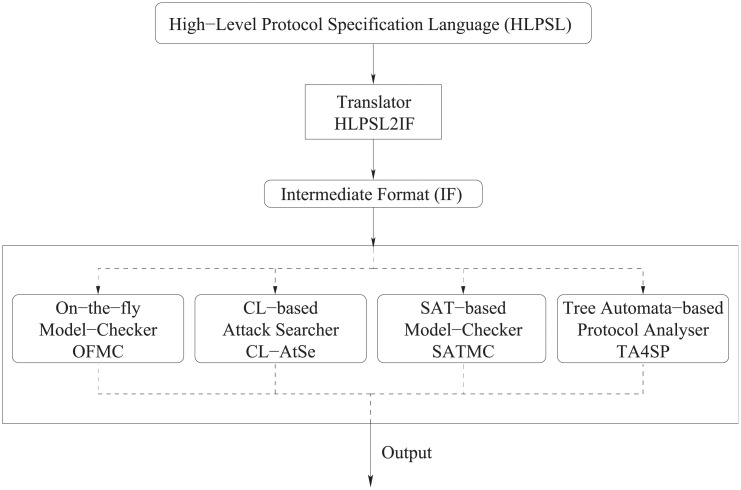
Architecture of the AVISPA Tool.

The AVISPA project aims at developing a push-button, industrial-strength technology for the analysis of large-scale Internet security-sensitive protocols and applications [26, 27]. The AVISPA Tool provides a suite of applications for building and analyzing formal models of security protocols [28, 29]. Protocol models are written in the High Level Protocol Specification Language (HLPSL). The structure of the AVISPA Tool is shown in Fig 16. A HLPSL specification is translated into the Intermediate Format (IF), using a translator called hlpsl2if. IF is a lower-level language than HLPSL and is read directly by the back-ends to the AVISPA Tool.

#### Simulation code development

The simulation code to verify our proposed scheme was implemented with six components.

1. Role of the mobile node: defines the role of the mobile node. In state 2, the identity and password are defined, and the secret property is set to verify these values are not exposed. In the registration phase, *CID*, *U* values are encrypted with the *SKmh* and transmitted because they communicate over a secure channel. In state 4, the mobile node receives registration parameters from the home node and proceeds with the login phase. At this time, the witness property is set to verify that the mobile node is securely shared with the home node for the *N*1. In state 8, it receives the message from the foreign node and the derived session key. Then, it verifies whether *N*2 and *N*3 values are securely shared and authenticated through the request property.
2. Role of the home node: defines the role of the home node. In state 3, the registration parameter is generated based on the received value from the mobile node, then transmitted through the secure channel. At this time, the secret property is set to verify whether the server’s secret key, *s*, is not exposed. In state 5, the home node receives a message from the foreign node in the authentication phase and generates a session key. Through the request property, it verifies that the *N*1 and *N*2 values created by the home node and the foreign node can be safely shared and authenticated. In addition, it verifies whether the *N*3 values can be safely shared and authenticated through the witness property. Finally, it verifies that the derived session key is not exposed through the secret property.
3. Role of the foreign node: defines the role of the foreign node. In state 1, a message is received from the mobile node in the login phase. Through the witness property, it verifies that *N*2 values can be safely shared and authenticated. In state 2, it receives the session key from the home node in the authentication phase. Through the secret property, it verifies whether the session key’s secrecy is exposed.
4. Role of session: defines the roles each node performs according to the defined role.
5. Role of environment: defines the attacker’s knowledge and attack environment. Here, the attacker can be a mobile node, home node, or foreign node. In addition, the attacker already knows the hash function and public information *ID*_*fn*_. A normal session with each party and three sessions where the party is replaced by the attacker are created.
6. Role of goal: verifies that the protocol designed for security properties (secret, request, witness) set in each role is safely satisfied.

All codes for proving the formal proof have been uploaded in figshare:https://doi.org/10.6084/m9.figshare.12624014.v1 [30]

#### Simulation result

AVISPA outputs the results as the following sections [31].

- SUMAMRY: indicates whether the protocol is safe, unsafe, or if the analysis is inconclusive.
- DETAILS: explains under what conditions the protocol is declared safe, or what conditions have been used for finding an attack, or finally why the analysis was inconclusive.
- PROTOCOL, GOAL, BACKEND: recall the name of the protocol, the goal of the analysis, and the name of the back-end used, respectively.

**Fig 17:**
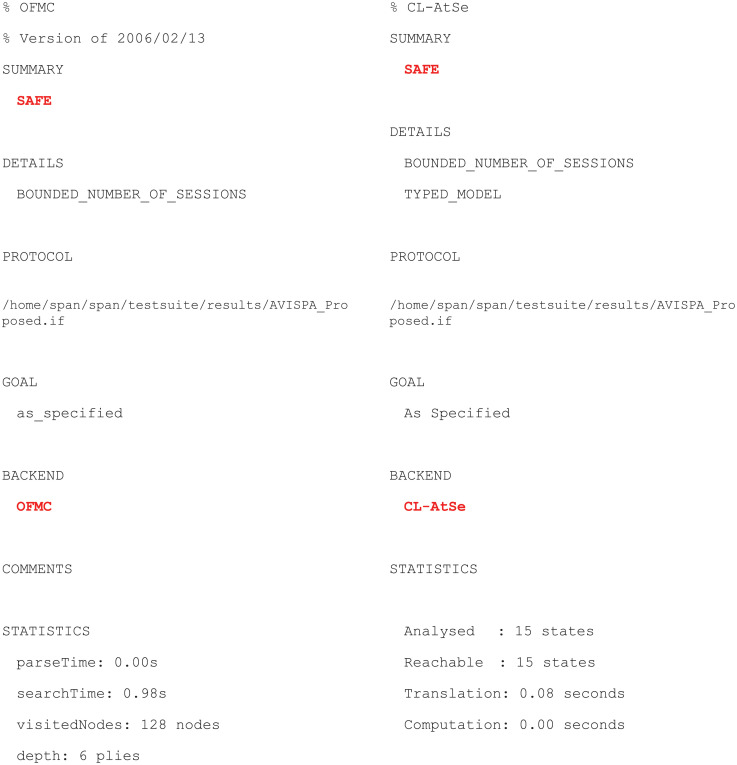
AVISPA simulation result for proposed scheme.

The proposed scheme was verified with the OFMC (On-the-fly Model-Checker) and CLAtSe (CL-based Attack Searcher) back-ends, and the results are displayed in Fig 17. We can see that OFMC and CLAtSe both found no attacks. In other words, the stated security goals were satisfied for a bounded number of sessions as specified in the role of environment. Furthermore, the results described previously indicate that our scheme is safe against man-in-the-middle and replay attacks under each back-end environment.

### 8.3 Privacy preserving analysis

This section proves the mathematical safety of the privacy conditions presented in Section 3.4, excluding user anonymity. User anonymity is trivial because *ID*_*MN*_ is not transmitted in the login request message of the proposed scheme. In proof, adversary A has the ability to control all communications, and A uses the following queries.

- Execute query: This query models a passive attack in which an attacker overhears a protocol *P* between player.
- Send query: This query receives the message generated by instance in response to another instance processing message *m* according to protocol *P*.
- Corrupt query: This query models the attacker’s corruption capabilities.

**Definition** Random oracle: In this paper, all participants and adversary A use a one-way hash function *h*(.), which is modeled as a *Hash* oracle. When a two-tuple (*x*, *y*) table of binary strings and a hash query are given, if *x* is discovered, the oracle returns *y*. Otherwise, it returns a uniformly random string *y*, and the (*x*, *y*) pair is stored in the corresponding table.

**Theorem** Let A be an adversary operating within polynomial time *t* for our protocol, P, in a random oracle. Let *D* be a uniformly distributed password and identity dictionary, and let *l* be the number of bits in a random nonce. The probability of *P*’s privacy security being broken by A is as follows:

$$\begin{array}{c} \frac{q_{hash}}{2^{\left|Hash\right|}}+\frac{q_{send}}{\sqrt{\pi \times 2^{|Hash|-1}}}+\frac{q_{hash}}{{\left|D\right|}^{2}\times 2^{l_{s}}}+\frac{q_{hash}}{\left(|D\right|\times 2^{l_{s}}{)}^{2}} \end{array}$$

where *q*_*hash*_, |*Hash*|, *q*_*send*_, |*D*|, and *l*_*s*_ denote the number of hash queries, the range space of the one-way hash function, the number of send queries, the size of *D*, and the secret parameter that determines the length of the random nonce, respectively.

**Proof**. The formal proof of our scheme consists of four different games *G*_*i*_ for *i* = 1, 2, 3, 4. Our protocol, P, runs from game *G*_1_ to game *G*_4_, and through a series of games, it will demonstrate that A has a negligible advantage in breaking user privacy.

**Game *G*_1_**: This game simulates an eavesdropping attack of an adversary A using the *Execute, Send, Hash* query. The attacker also queries the *Test* query and determines whether the result is a real session key *SK* or some other random value. Session key *SK* is computed as *SK* = *h*(*h*(*n*_1_)‖*h*(*CID*‖*s*)‖*h*(*n*_2_)‖*ID*_*FN*_). To compute the session key, A has to know *s*. Therefore, A cannot compute *h*(*CID*‖*s*) because *s* is a master secret key of *S*. Furthermore, A must know random nonce *n*_1_ and *n*_2_. Thus, the probability of adversary A winning this game through an eavesdropping attack is equal to the probability of correctly guessing the hash output value without any information and verifying the guessed session key with *V*_4_ through a hash query. Thus, we obtain the following:

$$\begin{array}{c} \frac{q_{hash}}{2^{\left|Hash\right|}} \end{array}$$ (1)

**Game *G*_2_**: In this game, adversary A models an active attack that sends a fake message to deceive the participants, using the *Send* and *Hash* queries. A can repeatedly generate hash queries to discover collisions. In our proposed scheme, *K*_3_ and *V*_3_ received to the home node are verified, and *V*_4_ received to the mobile node is verified. These three values are associated with random numbers *n*_1_, *n*_2_, *andn*_3_, respectively. Therefore, the messages are guaranteed to be random, and there is no collision while querying the *Send* oracle. Because the attacker cannot know the container of the hash, through a birthday attack [32], the attacker must find another input such that the hash value is the same.

$$\begin{array}{c} \frac{q_{send}}{\sqrt{\pi \times 2^{|Hash|-1}}} \end{array}$$ (2)

**Game *G*_3_**: This game simulates the *Corrupt*_*SC*_ oracle and models a lost smartcard attack. Adversary A can attempt a dictionary attack using information from a smartcard and can attempt to obtain an identity *ID*_*MN*_ and password *PW*_*MN*_. Thereafter, the adversary runs the experimental algorithm $EXP_{HASH,A}^{IDPWOBTAIN}$ to break user non-traceability. The success probability of $EXP_{HASH,A}^{IDPWOBTAIN}$ is represented by $EXP_{HASH,A}^{IDPWOBTAIN}=1]$. Therefore, the probability that A can guess the identity and password is $\frac{1}{\left|D\right|}$, and the probability that it can guess the random nonce is $\frac{1}{2^{l_{s}}}$. We obtain the following probability:

$$\begin{array}{c} \frac{q_{hash}}{{\left|D\right|}^{2}\times 2^{l_{s}}} \end{array}$$ (3)

**Algorithm 1**: $EXP_{HASH,A}^{IDPWOBTAIN}$

1 *Input*: *Corruput*_*SC*_ query’s output = *B*_*i*_, *R*_*i*_

2 *Output*: 0 or 1

3 Select each candidate for the identity($ID_{MN}^{\prime }$) and password($PW_{MN}^{\prime }$) from the dictionary.

4 Select the candidate of random nonce of length *l*_*s*_, *r*′

5 **if** $h(h(ID_{MN}^{\prime }\|r^{\prime })\|h(PW_{MN}^{\prime }\|r^{\prime }))=R_{i}$ **then**

6  Accept that the guessed identity and password are the actual user’s identity and password.

7  return 1

8 **else**

9  Reject that the guessed identity and password are the actual user’s identity and password.

10  return 0

11 **end**

**Game *G*_4_**: In this game, the attacker analyzes two different login request messages and verifies whether they are generated from the same mobile node. Suppose that the adversary extracts all parameters from the smartcard’s memory and overhears the communication message. Thereafter, the adversary runs the $EXP_{HASH,A}^{USERTRACE}$ experimental algorithm to break user non-traceability. The success probability of $EXP_{HASH,A}^{USERTRACE}$ is represented by $Pr\left[Succ_{4}\right]=Pr\left[EXP_{HASH,A}^{USERTRACE}=1\right]$. Using $EXP_{HASH,A}^{USERTRACE}$, if A is able to reveal the hash function, the adversary wins the game. However, computing the input value from the hash function is not computationally feasible because it has the following probability:

$$\begin{array}{c} \frac{q_{hash}}{\left(|D\right|\times 2^{l_{s}}{)}^{2}} \end{array}$$ (4)

**Algorithm 2**: $EXP_{HASH,A}^{USERTRACE}$

1 *Input*: two login messages

2 *Output*: 0 or 1

3 Eavesdrop login message {*CID*, *K*_2_, *K*_3_, *K*_4_, *T*_1_}

4 Eavesdrop another login message $\left\{CID^{\prime },K_{2}^{\prime },K_{3}^{\prime },K_{4}^{\prime },T_{1}^{\prime }\right\}$

5 Call the Reveal oracle. Let (*ID*_*MN*_, *r*) ← Reveal(*CID*) Call the Reveal oracle. Let ($ID_{MN}^{\prime },r^{\prime }$) ← Reveal(*CID*′) **if** $ID_{MN}^{\prime }=ID_{MN}$ **then**

6  Accept that eavesdropped login messages are created from the same mobile node

7  return 1

8 **else**

9  Accept that eavesdropped login messages are created from different mobile nodes

10  return 0

11 **end**

Combining Eqs (1)–(4), the results are as follows:

$$\begin{array}{c} \frac{q_{hash}}{2^{\left|Hash\right|}}+\frac{q_{send}}{\sqrt{\pi \times 2^{|Hash|-1}}}+\frac{q_{hash}}{{\left|D\right|}^{2}\times 2^{l_{s}}}+\frac{q_{hash}}{\left(|D\right|\times 2^{l_{s}}{)}^{2}} \end{array}$$

### 8.4 Informal security analysis

In this section, we perform an informal security analysis of our proposed scheme. The threat model is identical to the Dolev–Yao threat model applied to Ahmed et al.’s scheme. We considered not only the privacy preserving mentioned in Section 3.4, but also other security requirements to consider in the GLOMONET environment [33]. Table 2 compares privacy preserving, security attack resistance and some security requirements of our proposed scheme and other related schemes [4, 9, 11, 12, 34].

**Table 2:** Security comparison of the proposed scheme and other related schemes.

Property	Yoon	Mun	Lee	Gope	Ahmed	Proposed
*Pr1. User information related parameter privacy*	O	O	X	O	X	O
*Pr2. Mobile node anonymity*	O	O	O	O	O	O
*Pr3. Mobile node non-traceability*	X	X	O	O	X	O
*Pr4. Resistance to Mobile node impersonation*	O	X	X	O	X	O
*R1. Resistance to Replay attack*	O	X	X	O	O	O
*R2. Resistance to Privileged insider attack*	O	X	O	X	O	O
*R3. Resistance to Denial of service attack*	Δ^†1^	X	X	X	Δ^†1^	O
*R4. Resistance to Foreign bypass attack*	O	X	O	O	O	O
*R5. Resistance to Session key derived attack*	O	O	O	O	X	O
*R6. Forward secrecy*	X	O	X	X	O	O
*R7. Free and efficient password policy*	X	X	X	O	Δ^†2^	O
*R8. Session key agreement*	X	O	O	O	O	O
*R9. Mutual authentication*	O	X	Δ^†3^	O	Δ^†3^	O

^†1^ As only one of the user’s identity or password is checked in the login phase, a partial denial of service attack is possible.

^†2^ The mobile node can freely set the password, but the identity verification is omitted in the password change phase.

^†3^ No mutual authentication of the shared session key.

#### Perspective of Privacy Preserving

*Pr1*. *Offline identity/password guessing attack* To guess a mobile node’s identity or password, the adversary needs to know the hashed value of the user’s identity or password, such as *h*(*ID*) or *h*(*PW*), or the adversary already knows all the salted parameters. In our proposed scheme, the identity and password of the mobile node is transformed and stored by a random nonce *r*, such as *CID* = *h*(*ID*_*MN*_) ⊕ *r* and *U* = *h*(*PW*_*MN*_||*r*). During the login phase, a nonce *r* can only be derived by the correct identity of the mobile node. Therefore, an adversary that does not know the identity of the mobile node cannot derive the *r* value and consequently cannot infer the user’s password.

*Pr2*. *Mobile node anonymity* In our proposed scheme, *ID*_*MN*_, the identity of the mobile node, is stored as a *CID* in the form of pseudo-identity and transmitted in the login phase. To derive the user’s identity from *CID*, the adversary needs *r* and derives *h*(*ID*_*MN*_) and guesses *ID*_*MN*_ through identity guessing. Therefore, even if the adversary is eavesdropping on *CID* in advance, the adversary does not expose the mobile node’s identity, and the *CID* value also changes at the end of each session. Owing to this, our proposed scheme provides mobile node anonymity.

*Pr3*. *Mobile node non-traceability* To satisfy the non-traceability of the mobile node, the values of all messages transmitted from the mobile node in the login phase must be changed based on the random nonce in each session. If the contents of the message are the same, the attacker cannot specify the mobile node but can know whether different login request messages are sent from the same mobile node. In our proposed scheme, the values transmitted by the mobile nodes are *CID*, *K*_2_, *K*_3_, *K*_4_, and *T*_1_. Among them, *K*_2_, *K*_3_, and *K*_4_ are generated based on the random nonce *n*_1_ that is generated for each login phase. The value of *CID* is newly issued from the home node when the session is terminated once. That is, our proposed scheme satisfies non-traceability because the value of the login request message is replaced when the session ends and the next session begins.

**Fig 18:**
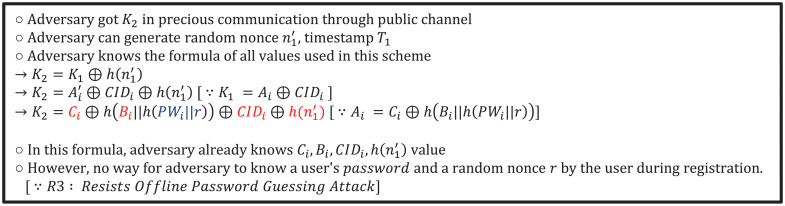
Resistance of proposed scheme to mobile mode impersonation attack.

*Pr4*. *Mobile node impersonation attack* To impersonate a mobile node, the adversary needs to create an *M*_1_. As *CID* is a value stored in the smartcard, the adversary can extract it from a stolen smartcard. Then, the adversary generates a random nonce $n_{1}^{\prime }$ and a current-time-based timestamp $T_{1}^{\prime }$. However, the adversary would not be able to infer a mobile node’s password or identity. As a result, the adversary would not be able to generate values for *K*_2_ and *K*_3_. As such, our proposed scheme can prevent mobile node impersonation attacks. Fig 18 provides a detailed proof of our proposed scheme’s resistance to a mobile node impersonation attack.

#### Perspective of security attack resistance and requirement

*R1*. *Resistance to replay attack* There is a timestamp in every message sent to the other party in our scheme, and a message is created based on the created timestamp. In other words, to resend the eavesdropping message, the timestamp must be updated to *T*′ as the time the adversary resends the message. However, to be successful in a replay attack, not only the timestamp but also other parameters created based on the timestamp need to be reprocessed. Therefore, our proposed scheme is safe from replay attack.

*R2*. *Resistance to privileged insider attack* In our proposed scheme, all values sent to the home node in the registration phase are processed by a random nonce *r*. In the case of a privileged insider of the home node, to guess the identity or password of the mobile node, the insider needs to know the value of *r* created in the mobile node. However, because the insider cannot derive the value of *r* with only *CID* and *U*, the identity and password cannot be guessed. Therefore, our proposed scheme is safe from privileged insider attack.

*R3*. *Resistance to denial of service attack* Denial of service attack is an attack that puts load on the home node by sending excessive login request messages to in the login phase. This attack often occurs when the user’s identity or password is not verified at the user’s end but is sent to the server and compared with the tables stored in the server’s database. Our proposed scheme verifies the mobile node’s identity and password at the smartcard and sends a login request message to the server when the value of $R_{i}^{\prime }$ obtained using the identity and password entered by the user is the same as the value of *R*_*i*_ in the smartcard.

*R4*. *Resistance to foreign bypass attack* The adversary can receive *M*_1_ from a mobile node and generate a message based on it to send to the home node, by impersonating as a foreign node. However, the adversary could not create parameter *V*_1_, because *V*_1_ is created with *FH*_*k*_, which is a pre-shared key between the foreign and home nodes. Therefore, our proposed scheme is safe from foreign bypass attacks.

*R5*. *Resistance to session key derived attack* In our proposed scheme, the parameters that make up the session key are *n*_1_, *A*_*i*_, *n*_2_, and *ID*_*FN*_. Among them, *ID*_*FN*_ is transmitted through the public channel, and thus the adversary can eavesdrop at any time. However, to obtain *A*_*i*_, the adversary needs to know the password and random nonce *r* of the mobile node. However, in **Pr1** we proved that neither of these could be derived by the adversary. As such, our proposed scheme is safe from session key derived attacks.

*R6*. *Forward secrecy* Forward secrecy means that each session key should not be related to the other session key created for the same user. In other words, even if the current session key is exposed by the adversary, the adversary should not be able to infer the next session key. In our scheme, the session key is generated based on the hash value of *n*_1_ generated by the mobile node and the hash value of *n*_2_ generated by the home node. These two random nonces are changed each time the mobile node starts a new session, and thus even if the existing session key is stolen, no information regarding the new session key can be obtained. Therefore, our proposed scheme satisfies forward secrecy.

*R7*. *Free and efficient password policy* Our proposed scheme provides a free and efficient password policy. In the registration phase, the mobile node user can choose their password without any restrictions. Furthermore, in the password change phase, the mobile node’s password can be changed by participating only in the mobile node without communicating with the server.

*R8*. *Session key agreement* Our proposed scheme generates the session key *SK* = *h*(*h*(*n*_1_)||*A*_*i*_||*h*(*n*_2_)||*ID*_*FN*_) after the home node validates the mobile node and foreign node in the authentication phase. The created session key is combined with *A*_*i*_ and *FH*_*k*_, which are known only to the mobile and foreign nodes. Then, the home node creates *H*_1_ and *H*_2_ such that only the legitimated party node can derive the session key. Then, the mobile node derives *h*(*n*_2_) through *A*_*i*_ and the foreign node derives *SK* through *FH*_*k*_ to establish session key agreement.

*R9*. *Mutual authentication* In the proposed scheme, the home node checks the validity of the mobile node through *K*_3_, and the validity of the foreign node through *V*_3_ derived via the pre-shared key *FH*_*k*_. In contrast, the session key generated by the home node is checked by the foreign node through $H_{3}=h(SK\|FH_{k}\|V_{3}^{\prime }\|T_{3})$, and the mobile node by *V*_4_ = *h*(*SK*|| *ID*_*FN*_||*T*_4_). Therefore, our proposed scheme satisfies mutual authentication.

## 9 Performance analysis

In this section, we compare and analyze the performance of our proposed scheme with other schemes in the existing GLOMONET environment. The criteria for performance are the computation cost and communication cost of each scheme, memory capacity of the smartcard and practical demonstration using NS2(Network Simulator 2).

### 9.1 Computational cost analysis

We examined how many cryptographic operations were performed during the registration phase and login, authentication phases in each node in similar environment. In order to measure the execution time for each protocol, first, compute computational overhead and measure the execution time of the cryptographic operations used in the protocol. Finally, substitute the measured time obtained. Notation for the execution time of each operation is given in Table 3.

**Table 3:** Notation for the execution time of each operation.

Notation	Definition
*T*_*h*_	Execution time for the hash function
$T_{Sym_{en}}$	Execution time for symmetric encryption
$T_{Sym_{de}}$	Execution time for symmetric decryption
$T_{ECC_{mul}}$	Execution time for Elliptic Curve Cryptosystem multiply
$T_{ECC_{en}}$	Execution time for Elliptic Curve Cryptosystem encryption
$T_{ECC_{de}}$	Execution time for Elliptic Curve Cryptosystem decryption
$T_{ECDSA_{sig}}$	Execution time for signing Elliptic Curve Cryptosystem signature (ECDSA)
$T_{ECDSA_{ver}}$	Execution time for verifying Elliptic Curve Cryptosystem signature (ECDSA)

**Fig 19:**
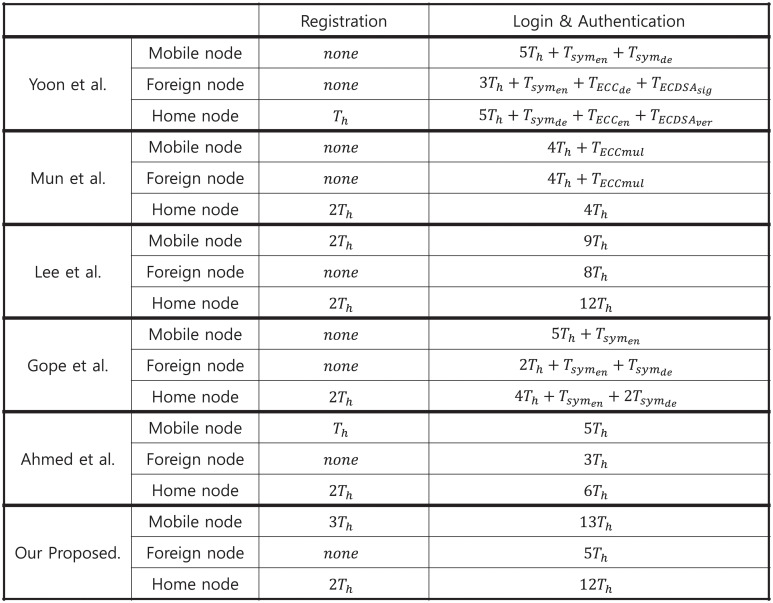
Computation time for each GLOMONET environment scheme.

In the registration phase, all the analyzed schemes had a similar number of hash function and random number generator operations. In the login and authentication phases, the operation types varied according to the encryption method used in each scheme. In the scheme of Yoon et al., Elliptic Curve Cryptosystem based encryption, decryption, and signature were used. In the scheme proposed by Mun et al., the Hash based Message Authentication Code (HMAC) and Elliptic Curve multiplication algorithm was used. In Gope et al.’s scheme, symmetric key encryption is used. In the schemes proposed by Lee et al., Ahmed et al., and our proposed scheme only used the hash function to reduce the scheme’s computational cost. The number of operations for each scheme and each node are listed in Fig 19.

In addition, we built our own test-bed to measure the execution time of each operation. We simulated the operations using Python cryptography library *hashlib*, *Crypto*, *ECIES*, *Pycoin* in the following hardware environment: Window 10 64bit, Intel Pentium CPU G4600, 3.60GHz, 16.0 GB RAM. We also assume that the hash function is implemented by SHA2, the symmetric encryption is implemented by AES-256 CTR Block Mode. Elliptic Curve Cryptosystem uses elliptic curve over the *secp*256*k*1 curve and implements 256-bit ECDSA signature, ECIES cryptosystem. Results of some computation time measurements for each operation time under the construction environment are given in Table 4.

**Table 4:** Computation time of each operation.

Notation	Computation Time (ms)
*T*_*h*_	0.038
$T_{Sym_{en}}$, $T_{Sym_{de}}$	0.997
$T_{ECC_{mul}}$	7.352
$T_{ECC_{en}}$	2.992
$T_{ECC_{de}}$	1.023
$T_{ECDSA_{sig}}$	56.819
$T_{ECDSA_{ver}}$	154.56

**Fig 20:**
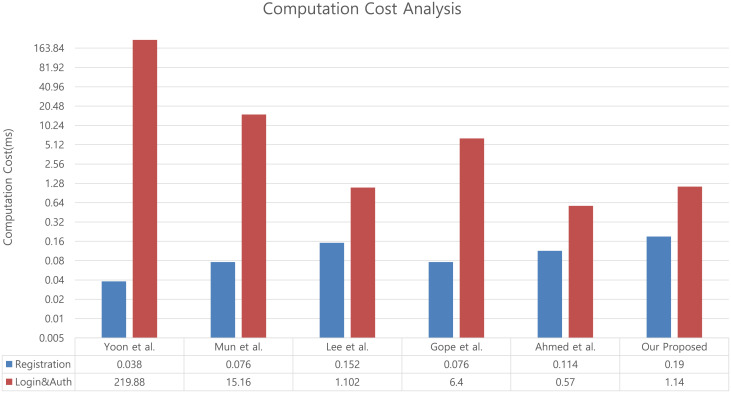
Computation cost analysis between proposed scheme and existing schemes.

Based on the results in Table 4, the computation cost required for our proposed scheme and existing scheme are displayed in Fig 20.

Our proposed scheme uses a lightweight authentication method that transmits secret information using bit-wise exclusive OR with information and verifies it through the hash function. The computation cost of the proposed scheme is approximately 1.5 to 2 times that of other lightweight authentication schemes. This is because we increased the number of hash functions to create a verification value to satisfy the session key mutual authentication and the number of random number generators to generate a new value for each mobile node login to satisfy the mobile node non-traceability. Therefore, this can be considered as the overhead to satisfy security requirements that were not included in the existing schemes.

### 9.2 Communication cost analysis

We compared and analyzed the size of the messages transmitted by each node in the registration, login, and authentication phases. We presume the size of identity/password, random nonce, and timestamp to be 128, 160, and 64 bits, respectively [35]. The Hash function and hash-based MAC have a 160-bit output size [36]. In an elliptic curve–based cryptosystem, the output size of the signature algorithm, the 256-bit ECDSA signature, is 512 bits and output size of the scalar multiplication operation is 360 bits [37, 38].

In existing papers, communication cost has been calculated assuming that the encrypted text had the same size regardless of the size of the plain text. However, this is practically impossible. Generally, the size of the ciphertext increases proportionally as the size of the plaintext increases. For more accurate communication cost comparison, we assume the symmetric key encryption method is AES-256, and the number of bytes of cipher text is set as follows according to the bytes of plain text [39].

$$\begin{array}{c} \left(\text{cipher}\;\text{text}\;\text{length})=16*\lfloor (\text{plain}\;\text{text}\;\text{length})/16+1\right\rfloor \end{array}$$

Further, the length of the ciphertext using elliptic curve integrated encryption scheme (ECIES) is typically twice that of the plain text [30, 40]. In addition, for X.509 certificates is set to 108 bytes according to standard document [41]. The above is summarized in Table 5 as follows:

**Table 5:** Communication cost(bit) of each operation.

Operation, Term	Communication cost(bit)
Identity, Password	128 bits
Random nonce	160 bits
Timestamp	64 bits
Hash function, HMAC	160 bits
ECDSA signature	512 bits
Elliptic Curve scalar multiplication	360 bits
AES Encryption (*k* bytes)	8 * 16 * ⌊ *k* /16 + 1⌋ bits
ECC Encryption (*k* bytes)	16*k* bits (2*k* bytes)
X.509 certificates	864 bits (108 bytes)
Track Sequence Number	64 bits

**Fig 21:**
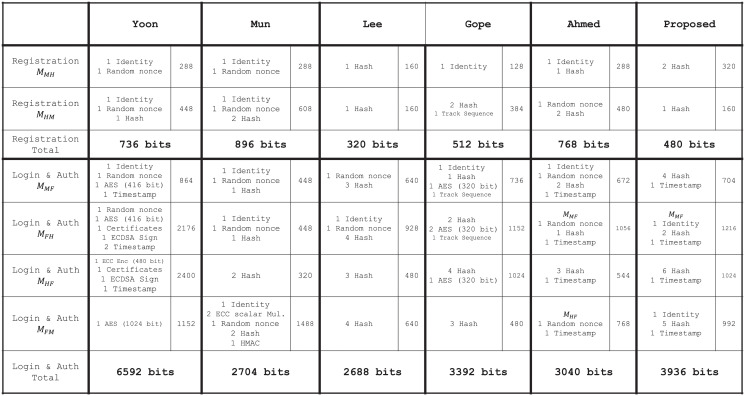
Detailed analysis by each communication message between our proposed scheme and existing scheme.

Based on this, the communication cost of each message is calculated by analyzing the composition of message of the existing scheme is as shown in Fig 21. In all schemes, in the registration phase, there are a total of 2 message exchanges between the Mobile Node and the Home Node, and in the Login & Authentication phase, a total of 4 messages are exchanged, 2 times each between the Mobile Node and Foreign Node, and the Foreign Node and Home Node. In the table, *M*_*AB*_ means the message transmitted from node *A* to node *B*. For example, *M*_*MH*_ is a message sent from Mobile Node to Home Node.

**Fig 22:**
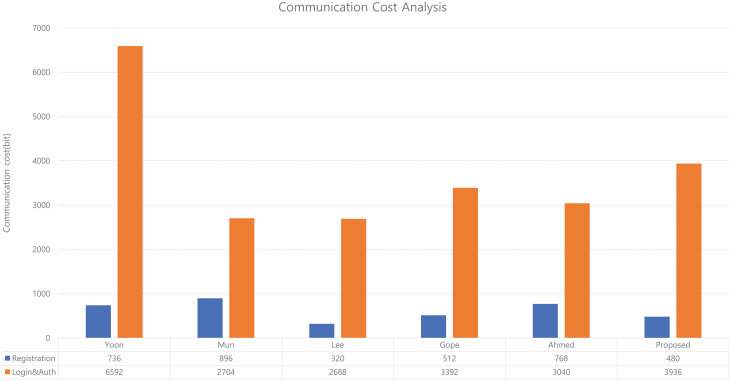
Communication cost analysis between our proposed scheme and existing scheme.

And the total communication cost required for our proposed scheme and existing scheme is as shown in Fig 22.

In our proposed scheme, the communication cost used in the registration phase is 480 bits, which is similar to the existing schemes. The communication cost used in the login/authentication phase is 3936 bits, which is slightly higher than that of other existing schemes. However, this can be regarded as an overhead that comes from increasing the verifying parameters to provide mutual authentication of session key and for update parameters to provide mobile node non-traceability that do not have existing schemes.

### 9.3 Memory capacity of the smartcard

**Fig 23:**
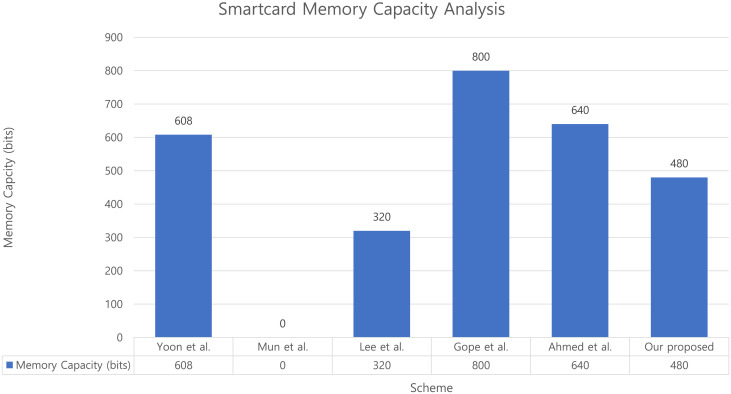
Comparison of memory capacity of the smartcard in our proposed scheme with those of existing schemes.

We also compared the memory capacity of the smartcard in our scheme with other associated schemes. We assume the output length of the parameter to be as shown in Table 5. In Yoon et al.’s scheme, a smartcard requires one identity, two random nonces, and one hash function output = (128 + 160 × 3 = 608) bits. In Mun et al.’s scheme, a smartcard is not issued. In Lee et al.’s scheme, one random nonce and 1 hash function = (160 × 2 = 320) bits are required. In Gope et al.’s scheme, the smartcard requires one random nonce and four hash functions = (160 × 5 = 800) bits. In Ahmed et al.’s scheme, the smartcard requires one random nonce and three hash functions = (160 × 4 = 640) bits. Finally, in our proposed scheme, the smartcard requires only three hash functions (160 × 3 = 480) bits. In conclusion, Fig 23 presents a comparison of the memory capacity of the smartcard. Even though our scheme needs slightly more memory capacity for the smartcard than existing schemes, our scheme can guarantee safety against various existing attacks and preserves privacy, as shown in Table 2.

### 9.4 Practical demonstration

The proposed scheme is simulated using the widely accepted NS2 simulator tool to provide a practical perspective. NS2 is an event-driven simulation tool that has proven useful in studying the dynamic nature of communication networks [42].

#### 9.4.1 Simulation environment

The operating system used in the NS2 simulation is Ubuntu 14.04 LTS, and the NS2 version used is ns-2.35 allinone [43]. We have defined scenarios that will simulate the proposed GLOMONET authentication protocol as follows.

1. Four foreign nodes (FNs) are communicating with the home node (HN), and each neighboring pair is separated by a distance of 100 *m*.
2. Between the mobile node and FN, a wireless environment is used, and between the HN and FN, a wired environment is used.
3. The mobile nodes are restricted within a 500 × 500*m*^2^ area with a speed of 2*m*/*s*.
4. For each scenario, 10, 20, 30, and 40 mobile nodes are deployed.
5. The proposed GLOMONET is simulated for 60 s, and each mobile node sends a login request message once every 1 s.

**Fig 24:**
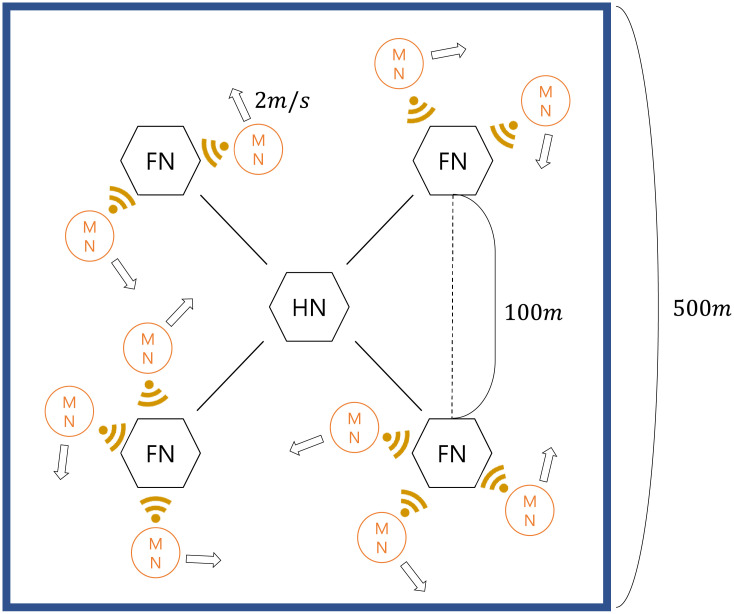
Topology of our simulation environment (when there are 10 mobile nodes).

As described in Section 8.2, the proposed scheme consists of four messages between the mobile node, FN, and HN in the login and authentication phase with TCP communication. Login request message *M*_*MF*_ has a size of 704 bits, and the authentication request and response message *M*_*FH*_, *M*_*HF*_, *M*_*FM*_ have sizes of 704, 1216, 1024, and 992 bits, respectively. Fig 24 depicts our simulation environment topology.

#### 9.4.2 Simulation results

To measure the performance of the proposed authentication protocol, we calculated the throughput, packet loss ratio, and end-to-end delay (EED), which are important indicators of the network performance. The calculation formula for each indicator is as follows.

1. Throughput: $\frac{N_{r}\times \left|Packet\right|}{T_{s}}$
2. Packet Loss Ratio: $1-\frac{N_{r}}{N_{s}}$
3. End-to-End Delay: $\sum_{k=1}^{N_{p}}\left(T_{r_{k}}-T_{s_{k}}\right)/N_{p}$

Here, *N*_*r*_ is the number of received packets, *N*_*s*_ is the number of sent packets, |*Packet*| is the bit size of the packet, *T*_*s*_ is the total simulation time, *N*_*p*_ is the number of total packets, $T_{r_{k}}$ is the *k*-th packet’s received time, and $T_{s_{k}}$ is the *k*-th packet’s sent time.

#### 9.4.3 Impact on throughput

**Fig 25:**
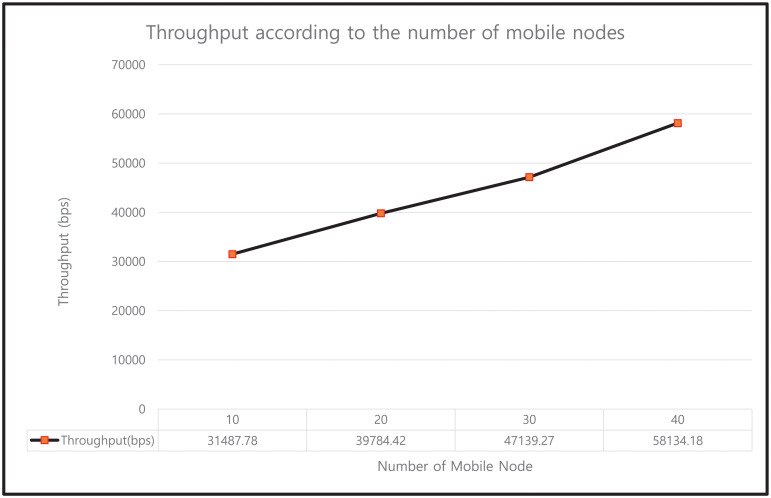
Throughput according to the number of mobile nodes.

Throughput is defined as the number of bits transmitted per unit time. The throughput (in bps) values for our protocol under different mobile nodes, as plotted in Fig 25, are 31487.78, 39784.42, 47139.27, and 58134.18 bps for the 10, 20, 30, and 40 mobile nodes, respectively. The results indicate that as the number of mobile nodes increases, the number of messages exchanged per unit time increases; thus, it can be observed that the throughput increases linearly with the number of mobile nodes.

#### 9.4.4 Impact on packet loss ratio

**Fig 26:**
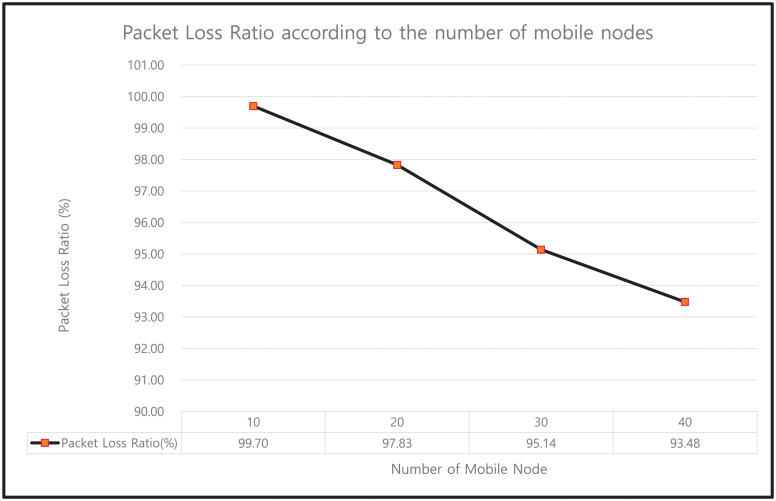
Packet loss ratio according to the number of mobile nodes.

The packet loss ratio is defined as the ratio of the total number of data packets lost at the destination and the total number of sent packets. The packet loss ratio (in %) values for our protocol under different mobile nodes, as plotted in Fig 26, are 99.70, 97.83, 95.14, and 93.48% for 10, 20, 30, and 40 mobile nodes, respectively. The results indicate that as the number of mobile nodes increases, the network has more congestion. Moreover, if the mobile node is far from the HN, then the energy in a sent packet will be dry and dropped at the FN.

#### 9.4.5 Impact on End-to-End Delay

**Fig 27:**
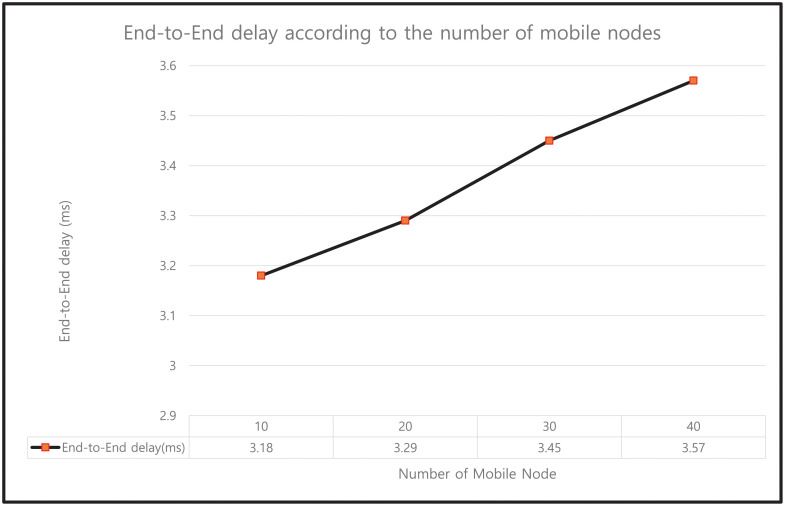
End-to-end delay according to the number of mobile nodes.

EED is defined as the time taken for a packet to be sent across a network. The EED (in ms) values for our protocol under different mobile nodes, as plotted in Fig 27, are 3.18, 3.29, 3.45, and 3.57 ms for 10, 20, 30, and 40 mobile nodes, respectively. The results indicate that as the number of mobile nodes increases, the maximum flow of messages results in higher distances and more bottlenecks in the network.

## 10 Conclusion

Owing to the development of mobile devices, several services are accessible to us regardless of time and place. Roaming is used in the GLOMONET environment to use the services of the home network in other places. In this environment, numerous authentication schemes have been proposed to securely share the session key between the home node and mobile node through the foreign node. Ahmed et al. proposed a lightweight user authentication scheme that uses only hash, bit-wise exclusive, or concatenation operations without any encryption method. However, we found that their scheme is incomplete and vulnerable to multiple attacks. Accordingly, we proposed a new lightweight scheme that compensates for existing security vulnerabilities such as user traceability and identity/password guessing attacks and preserves user privacy. The proposed scheme was proven to be safe using ProVerif and AVISPA, which are formal security analysis methods. Furthermore, via informal security analysis, we demonstrated the proposed scheme can defend against attacks that can be implemented in a user authentication.

## Supporting information

S1 Data (ZIP)
